# A shared neural basis underlying psychiatric comorbidity

**DOI:** 10.1038/s41591-023-02317-4

**Published:** 2023-04-24

**Authors:** Chao Xie, Shitong Xiang, Chun Shen, Xuerui Peng, Jujiao Kang, Yuzhu Li, Wei Cheng, Shiqi He, Marina Bobou, M. John Broulidakis, Betteke Maria van Noort, Zuo Zhang, Lauren Robinson, Nilakshi Vaidya, Jeanne Winterer, Yuning Zhang, Sinead King, Tobias Banaschewski, Gareth J. Barker, Arun L. W. Bokde, Uli Bromberg, Christian Büchel, Herta Flor, Antoine Grigis, Hugh Garavan, Penny Gowland, Andreas Heinz, Bernd Ittermann, Hervé Lemaître, Jean-Luc Martinot, Marie-Laure Paillère Martinot, Frauke Nees, Dimitri Papadopoulos Orfanos, Tomáš Paus, Luise Poustka, Juliane H. Fröhner, Ulrike Schmidt, Julia Sinclair, Michael N. Smolka, Argyris Stringaris, Henrik Walter, Robert Whelan, Sylvane Desrivières, Barbara J. Sahakian, Trevor W. Robbins, Gunter Schumann, Tianye Jia, Jianfeng Feng, IMAGEN Consortium, STRATIFY/ESTRA Consortium, Betteke Maria van Noort, ZIB Consortium

**Affiliations:** 1 Institute of Science and Technology for Brain-Inspired Intelligence, Fudan University, Shanghai, China; 2 Key Laboratory of Computational Neuroscience and Brain-Inspired Intelligence (Fudan University), Ministry of Education, Shanghai, China; 3 Faculty of Psychology, Technische Universität Dresden, Dresden, Germany; 4 School of Health Sciences, The University of Manchester, Manchester, UK; 5 Social Genetic and Developmental Psychiatry Centre, Institute of Psychiatry, Psychology and Neuroscience, King’s College London, London, UK; 6 Clinical and Experimental Sciences, Faculty of Medicine, University of Southampton, Southampton, UK; 7 Department of Psychology, MSB Medical School Berlin, Berlin, Germany; 8 Department of Psychological Medicine, Section for Eating Disorders, Institute of Psychiatry, Psychology and Neuroscience, King’s College London, London, UK; 9 South London and Maudsley NHS Foundation Trust, London, UK; 10 Department of Psychiatry and Neurosciences, Charité–Universitätsmedizin Berlin, corporate member of Freie Universität Berlin, Humboldt-Universität zu Berlin, and Berlin Institute of Health, Berlin, Germany; 11 Department of Education and Psychology, Freie Universität Berlin, Berlin, Germany; 12 Psychology Department, University of Southampton, Southampton, UK; 13 School of Medicine, Center for Neuroimaging, Cognition and Genomics, National University of Ireland (NUI) Galway, Galway, Ireland; 14 Department of Child and Adolescent Psychiatry and Psychotherapy, Central Institute of Mental Health, Medical Faculty Mannheim, Heidelberg University, Mannheim, Germany; 15 Department of Neuroimaging, Institute of Psychiatry, Psychology and Neuroscience, King’s College London, London, UK; 16 Discipline of Psychiatry, School of Medicine and Trinity College Institute of Neuroscience, Trinity College Dublin, Dublin, Ireland; 17 University Medical Centre Hamburg-Eppendorf, Hamburg, Germany; 18 Institute of Cognitive and Clinical Neuroscience, Central Institute of Mental Health, Medical Faculty Mannheim, Heidelberg University, Mannheim, Germany; 19 Department of Psychology, School of Social Sciences, University of Mannheim, Mannheim, Germany; 20 NeuroSpin, C.E.A., Université Paris-Saclay, Gif-sur-Yvette, France; 21 Departments of Psychiatry and Psychology, University of Vermont, Burlington, VT USA; 22 Sir Peter Mansfield Imaging Centre School of Physics and Astronomy, University of Nottingham, Nottingham, UK; 23 Physikalisch-Technische Bundesanstalt (PTB), Braunschweig and Berlin, Germany; 24 Institut des Maladies Neurodégénératives, UMR 5293, CNRS, CEA, Université de Bordeaux, Bordeaux, France; 25 Institut National de la Santé et de la Recherche Médicale, INSERM U1299 ‘Trajectoires développementales en psychiatrie’, Université Paris-Saclay, Ecole Normale supérieure Paris-Saclay, CNRS UMR9010, Centre Borelli, Gif-sur-Yvette, France; 26 AP-HP, Sorbonne Université, Department of Child and Adolescent Psychiatry, Pitié-Salpêtrière Hospital, Paris, France; 27 Institute of Medical Psychology and Medical Sociology, University Medical Center Schleswig-Holstein, Kiel University, Kiel, Germany; 28 Department of Psychiatry and Neuroscience and Centre Hospitalier Universitaire Sainte-Justine, University of Montreal, Quebec, Canada; 29 Department of Child and Adolescent Psychiatry and Psychotherapy, University Medical Centre Göttingen, Göttingen, Germany; 30 Department of Psychiatry and Neuroimaging Center, Technische Universität Dresden, Dresden, Germany; 31 Division of Psychiatry and Department of Clinical, Educational & Health Psychology, University College London, London, UK; 32 School of Psychology and Global Brain Health Institute, Trinity College Dublin, Dublin, Ireland; 33 Department of Psychiatry and Behavioural and Clinical Neuroscience Institute, University of Cambridge, Cambridge, UK; 34 Department of Psychology and Behavioural and Clinical Neuroscience Institute, University of Cambridge, Cambridge, UK; 35 Department of Sports and Health Sciences, University of Potsdam, Potsdam, Germany; 36 PONS Centre, Institute for Science and Technology of Brain-inspired Intelligence (ISTBI), Fudan University, Shanghai, China; 37 School of Mathematical Sciences and Centre for Computational Systems Biology, Fudan University, Shanghai, China; 38 Department of Computer Science, University of Warwick, Coventry, UK; 39 Fudan ISTBI–ZJNU Algorithm Centre for Brain-inspired Intelligence, Zhejiang Normal University, Jinhua, China

**Keywords:** Psychiatric disorders, Cognitive control

## Abstract

Recent studies proposed a general psychopathology factor underlying common comorbidities among psychiatric disorders. However, its neurobiological mechanisms and generalizability remain elusive. In this study, we used a large longitudinal neuroimaging cohort from adolescence to young adulthood (IMAGEN) to define a neuropsychopathological (NP) factor across externalizing and internalizing symptoms using multitask connectomes. We demonstrate that this NP factor might represent a unified, genetically determined, delayed development of the prefrontal cortex that further leads to poor executive function. We also show this NP factor to be reproducible in multiple developmental periods, from preadolescence to early adulthood, and generalizable to the resting-state connectome and clinical samples (the ADHD-200 Sample and the STRATIFY & ESTRA Project). In conclusion, we identify a reproducible and general neural basis underlying symptoms of multiple mental health disorders, bridging multidimensional evidence from behavioral, neuroimaging and genetic substrates. These findings may help to develop new therapeutic interventions for psychiatric comorbidities.

## Abstract (web summary)

Evidence from large longitudinal neuroimaging cohorts, which include genetic and behavioral data, suggest a common neural basis for symptoms seen across multiple psychiatric disorders.

## Main

The coexistence of multiple psychiatric conditions, known as psychiatric comorbidity^1^, has garnered substantial attention due to its high prevalence and long-lasting impact^2^. Individuals with comorbid psychiatric diagnoses often experience poorer outcomes and severe deficits in various cognitive and behavioral domains^3^. Notably, many psychiatric disorders, for example, externalizing and internalizing disorders, have their approximate peak onset in adolescence, coinciding with the emergence of comorbidity^4,5^. For instance, a population-based study on the well-being of adolescents found that 27.9% of participants aged 14–17 reached multiple diagnostic criteria^6^. The high prevalence of comorbid mental disorders suggests shared neurobiological origins among different psychopathologies^2^. However, the neuropsychopathological mechanism of psychiatric comorbidity, particularly during the critical period of adolescence, remains elusive.

Recently, emerging evidence has suggested a general psychopathology factor (that is, the p factor) underlying higher vulnerability for different psychiatric disorders^7^. Statistically, the p factor summarizes a pattern of positive correlations among symptoms; however, it leaves no room for alternative latent effects (for example, anticorrelation among symptoms). Indeed, it was argued that the behavioral p factor is largely equivalent to a sum of all symptoms^8^. Further, previous neuroimaging studies that investigated the neural correlates of the p factor mainly relied on task-free modalities, such as resting state^9^, diffusion^10^ and structural magnetic resonance imaging (MRI)^11^. However, although these task-free neural correlates of this p factor represent varied neurobiological information, they do not aid in specifying the neurocognitive processes underlying multiple psychopathologies^12,13^. Instead, the relevant cognitive brain circuitry can be mapped using multitask functional MRI (fMRI) data, which have also been used previously to identify circuit-specific neural signatures of externalizing symptoms^14^.

In contrast, crossdisorder genetic studies further revealed that many psychiatric disorders share high degrees of positive genetic correlations^15,16^, and the common genetic variants predominantly involved neurodevelopmental processes^17,18^. However, opposite genetic effects were also identified among psychiatric disorders^17^, which further highlighted the complexity of shared biological processes across multiple mental disorders. Therefore, it is necessary to integrate behavioral, neuroimaging and genetic evidence to establish coherent neurobiological crossdisorder neural factors (that is, the NP factor) that are not only shared among different psychopathologies but could also be attributed to specific cognitive brain circuits and genetic variants.

Notably, mounting evidence propose that many mental disorders can be understood as extreme deviations from a continuous spectrum in the population and different mental disorders may demonstrate similar deficits in multiple cognitive functions, as envisaged by Research Domain Criteria^19^. This new understanding inspires us to investigate potential transdiagnostic neurobiological processes from population-based data that are enriched with task-based fMRIs of multiple cognitive domains and symptom measurements covering a wide range of mental disorders. This approach also allows us to avoid the dilemma of case–control studies in identifying transdiagnostic biomarkers, where comorbidity is generally considered a major confounding factor to be removed. Furthermore, considering the replication crisis in neuroimaging studies, it was suggested that combining large neuroimaging samples and machine-learning approaches (that is, combining training and validation processes) could increase the reliability and reproducibility of identified neurobiomarkers^20,21^.

**Fig. 1:**
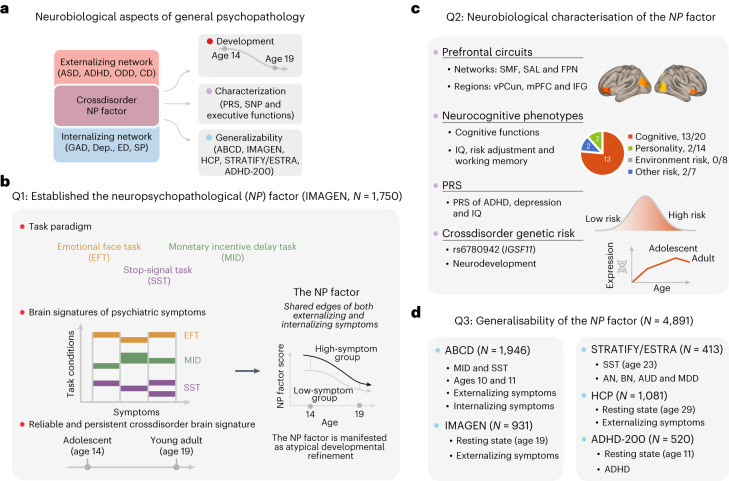
Overview of research questions and analyses. **a**, This study aims to answer three questions (Q1–Q3) about multiple neurobiological aspects of general psychopathology. **b**, We identified the NP factor in the IMAGEN dataset at ages 14 and 19 on the basis of task-based FC with a CPM (Q1; *N* = 1,750). **c**, We characterized the NP factor using multiple neurocognitive behaviors and genetic substrates (Q2). **d**, We checked the generalizability of the NP factor in multiple developmental periods using different fMRI states (Q3; *N* = 4,942). AN, anorexia nervosa; BN, bulimia nervosa; AUD, alcohol use disorder ; MDD, major depressive disorder; ADHD, attention-deficit/hyperactivity disorder; ASD, autism spectrum disorder; CD, conduct disorder; ODD, oppositional defiant disorder; GAD, general anxiety disorder; Dep., depression; ED, eating disorder; SP, specific phobia; FPN, frontoparietal network; IFG, inferior frontal gyrus; mPFC, medial prefrontal cortex; SAL, salience network; SMF, superior medial frontal network; NP factor score, the connectivity strength of the NP factor; vPCun, ventral precuneus.

In this study, we will address the following three specific major questions regarding the shared neural bases of behavioral symptoms related to psychiatric disorders (Fig. 1a): (1) Can we establish an NP factor underlying both externalizing and internalizing symptoms on the basis of the multiple task-based connectomes of fMRI? (2) Is the NP factor supported by genetic and neurobehavioral substrates of comorbid mental disorders? (3) Could the NP factor be generalized to other developmental stages and clinical crossdisorder datasets?

## Results

### Summary of major analytic steps

**Fig. 2:**
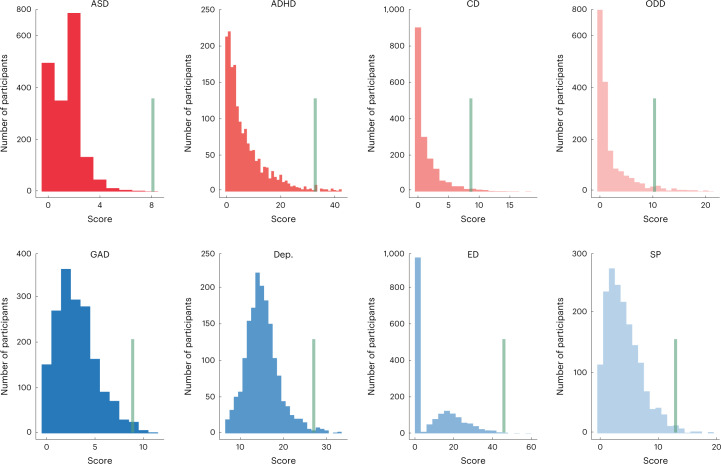
Histograms of externalizing and internalizing symptoms at age 14. Externalizing symptoms include ASD, ADHD, CD and ODD; internalizing symptoms include GAD, depression, ED and SP. The green line in each graph marks an approximate threshold for individuals who are high risk (that is, chance with diagnoses is over 50% according to Development And Well-Being Assessment (DAWBA)).

**Extended Data Fig. 1:**
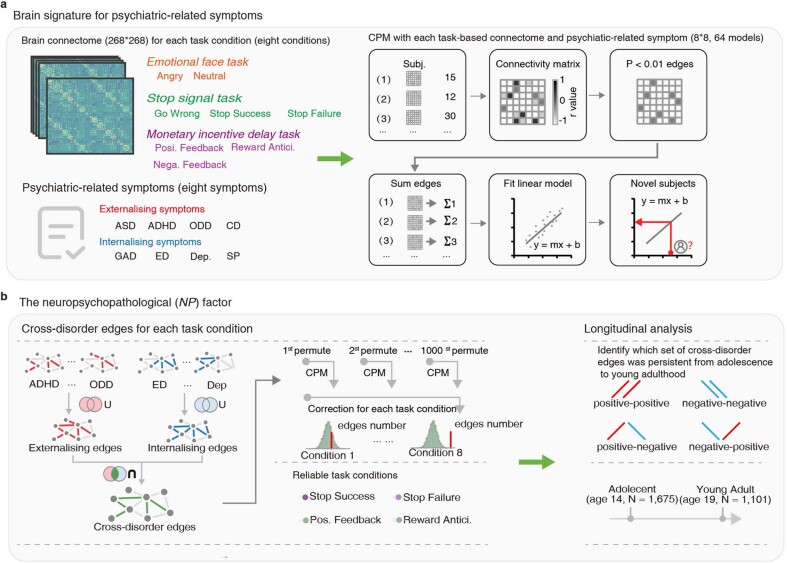
The construction process of the NP factor. a. For each participant, we first constructed the brain connectome for each task condition of the three tasks with a whole-brain 268 region atlas. Specifically, the EFT contained angry and neutral conditions; the SST contained go wrong, stop success, and stop failure conditions; the MID task contained positive feedback, reward anticipation, and negative feedback conditions. We also collected eight behavioural symptoms: four externalising symptoms (ASD, ADHD, ODD, and CD) and four internalising symptoms (GAD, ED, Dep. And SP). We then estimated the brain signature for each behavioural symptom with each task-based connectome by the machine-learning method of Connectome-based predictive modeling (CPM). b. With the identified brain signature for behavioural symptoms, we next constructed the Neuropsychopathological (NP) Factor in three steps. First, for each task condition, we counted the number of cross-disorder edges that the edge was predictive of both externalising and internalising symptoms. Then we used the permutation test to identify reliable conditions where the number of cross-disorder edges was significantly higher than random discovery. These reliable cross-disorder edges were then divided into four groups regarding their simultaneous predictive effects for externalising and internalising symptoms (that is positive-positive, positive-negative, negative-positive and negative-negative), and we conducted longitudinal analyses to identify which groups of cross-disorder edges could be used to form the NP factor that is still predictive to both externalising and internalising symptoms at age 19.

First, we leveraged the population-based IMAGEN cohort (aged 14 years, *N* = 1,750, 882 girls; Extended Data Table 1) to estimate the brain signature of eight behavioral symptoms using the connectivity-based predictive model grounded on multiple task-based fMRIs (Fig. 1b). Specifically, we estimated the condition-specific functional connectivity (FC) using a well-established whole-brain functional atlas^22^. These task-based FCs were used in a connectome-based predictive model (CPM)^23^ to predict each of the eight behavioral symptoms related to psychiatric disorders (Fig. 2 and Supplementary Table 1). First, in the CPM, univariately significant FCs were used to establish a linear model for prediction, and crossvalidation was implemented to avoid overfitting and improve the model’s reproducibility in novel samples^24^ (Extended Data Fig. 1a). Second, we conducted longitudinal analyses to identify a sustainable transdiagnostic NP factor that was predictive of both externalizing and internalizing symptoms across adolescence and early adulthood (Fig. 1b and Extended Data Fig. 1b). Third, we characterized the NP factor in multiple neurobiological aspects (Fig. 1c), including its neuroanatomical interpretation (that is, the brain networks involved), neurobehavioral relevance (with the corresponding task performance) and its associations with common environmental and behavioral risk factors. We also investigated candidate biological processes and genetic substrates underlying the crossdisorder NP factor. Finally, we assessed and confirmed the generalizability of the NP factor in other developmental periods and resting-state MRIs (from Adolescent Brain Cognitive Development (ABCD) and Human Connectome Project (HCP) cohorts) and in clinical datasets (from the ADHD-200 Sample (ADHD-200) and the STRATIFY/ESTRA Project (STRATIFY/ESTRA)) (Fig. 1d).

### Brain signatures of externalizing and internalizing symptoms

**Fig. 3:**
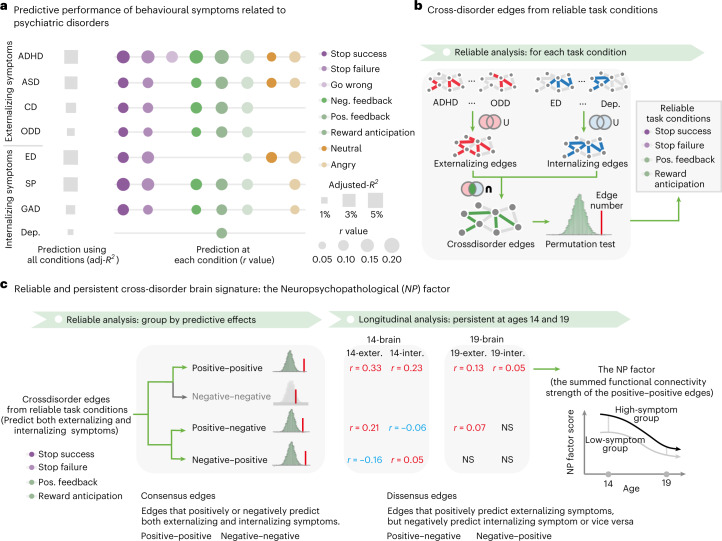
Identification of the NP factor. **a**, The predictive performance of behavioral symptoms related to psychiatric disorders using the task-based connectivity model. Task-based connectivity was obtained from the EFT (angry and neutral conditions), the MID task (reward anticipation, positive reward feedback and negative reward feedback conditions) and the SST (go-wrong, stop-success and stop-failure conditions). Overall predictive performance was estimated using a multiple regression model with predicted symptoms of all task conditions. **b**, Crossdisorder edges could predict externalizing and internalizing symptoms simultaneously. Externalizing symptoms consisted of ASD, ADHD, CD and ODD. Internalizing symptoms comprised GAD, Dep., ED and SP. For each task condition, we further estimated whether the set of crossdisorder edges was significantly larger than a random discovery with permutation tests. The results showed that only conditions from the SST and MID task had significantly more crossdisorder edges than a random observation. **c**, Using reliability and longitudinal analyses, we identified the NP factor that was positively predictive for both externalizing and internalizing symptoms across ages 14 and 19. 14-brain, brain at age 14; 19-brain, brain at age 19; exter., externalizing; inter., internalizing; neg., negative; NP factor score, the summed FC strength of the transdiagnostic edges; NS, not significant; pos., positive.

We found the task-based FC derived from the eight task conditions significantly predicted most behavioral symptoms after Bonferroni correction (Fig. 3a and Supplementary Table 2). To evaluate the integrated predictive effects, we used a multiple regression model combining the predicted symptom scores of different task conditions. Specifically, externalizing symptoms of attention-deficit/hyperactivity disorder (ADHD; adjusted *R*^2^ (adj-*R*^2^) = 4.28%), autism spectrum disorder (ASD; adj-*R*^2^ = 2.66%), conduct disorder (CD; adj-*R*^2^ = 2.23%) and oppositional defiant disorder (ODD; adj-*R*^2^ = 1.30%) were largely explained by the cognitive domains of reward sensitivity and inhibitory control (Supplementary Table 3). Similarly, three internalizing symptoms (specific phobia (SP), generalized anxiety disorder (GAD) and eating disorder (ED)) were also significantly predicted by reward sensitivity, inhibitory control and emotional reactivity (SP adj-*R*^2^ = 4.83 %, GAD adj-*R*^2^ = 1.97%, ED adj-*R*^2^ = 4.89%; Supplementary Table 3). Our results suggested that we could characterize the shared neural configurations underlying general psychopathology using these brain signatures of externalizing and internalizing symptoms.

### Construction of a reliable and persistent NP factor

Next, we aimed to establish the NP factor, consisting of crossdisorder edges, in relation to both externalizing and internalizing symptoms (Fig. 3b). The NP factor needed to meet two additional criteria: (1) only crossdisorder edges from the most reliable and informative task conditions should be used to construct the NP factor; and (2) the NP factor should be a persistent predictor of different behavioral symptoms from adolescence to young adulthood, given the persistent nature of psychiatric comorbidity over time^3,4^.

First, we investigated the enrichment of crossdisorder edges using permutation tests to evaluate if the number of crossdisorder edges (*n*_edge_) identified in a given task condition was significantly larger than that in a random discovery. In each permutation iteration, we reconducted the previously mentioned CPM process using randomly reshuffled participant labels and counted the corresponding number of crossdisorder edges (Fig. 3b, see Methods for details). We found that only conditions from the stop signal task (SST) and the monetary incentive delay (MID) task had significantly more crossdisorder edges than in a random observation (for SST, stop success *n*_edge_ = 325, stop failure *n*_edge_ = 297, positive feedback *n*_edge_ = 344; for MID, reward anticipation *n*_edge_ = 316; all *P* values based on permutation (*P*_perm_) < 0.001; Supplementary Table 4). These four task conditions were therefore considered reliable and included in the following analyses. Next, to improve interpretability, we then stratified crossdisorder edges from the four reliable task conditions into four groups in terms of their predictive effects (Fig. 3c), that is, positive–positive (*n*_edge_ = 136) and negative–negative (*n*_edge_ = 64) consensus edges (which showed positive or negative correlations with both externalizing and internalizing symptoms simultaneously), and positive–negative (*n*_edge_ = 1,032) and negative–positive (*n*_edge_ = 48) dissensus edges (which had opposite correlations with externalizing and internalizing symptoms). The number of positive–negative edges (*P*_perm_ < 0.001), negative–positive edges (*P*_perm_ = 0.002) and positive–positive edges (*P*_perm_ < 0.001) were significantly higher than that in random discoveries (Supplementary Table 5a), and were therefore included in the following analyses.

Finally, we examined the longitudinal consistency for each of these three crossdisorder edge groups and characterized the summed FC strength of the longitudinally consistent crossdisorder edge group as the NP factor underlying externalizing and internalizing disorders simultaneously (Fig. 3c). We found that only the summed FC strength of positive–positive consensus edges was associated with externalizing and internalizing symptoms simultaneously at both ages 14 (for externalizing symptoms, *N* = 1,724, *r* = 0.31, 95% confidence interval (CI) = [0.27,∞), one-tailed *P*_perm_ < 0.001; for internalizing symptoms, *r* = 0.23, 95% CI = [0.19,∞), one-tailed *P*_perm_ < 0.001; because the symptom-predictive model was trained at age 14, *P* values here were estimated using permutation tests that are robust to overfitting; Supplementary Table 5c) and 19 (for externalizing symptoms, *N* = 1,101, *r* = 0.13, 95% CI = [0.08,∞), *t*-statistic (*t*) = 4.43, *P*_one-tailed_ = 1.51 × 10^−5^; for internalizing symptoms, *r* = 0.051, 95% CI = [0.001,∞), *t* = 1.70, *P*_one-tailed_ = 0.044; Supplementary Table 6a). Moreover, the summed FC strength of positive–positive edges at age 14 could also predict the subsequent behavioral symptoms measured at age 19 (for externalizing symptoms, *N* = 1,045, *r* = 0.13, 95% CI = [0.08,∞), *t* = 4.26, *P*_one-tailed_ = 1.24 × 10^−5^; for internalizing symptoms, *r* = 0.17, 95% CI = [0.12,∞), *t* = 5.54, *P*_one-tailed_ = 1.60 × 10^−8^; Supplementary Table 6b), even after controlling for the baseline measurements (for externalizing symptoms, *N* = 1,036, *r* = 0.073, 95% CI = [0.02,∞), *t* = 2.34, *P*_one-tailed_ = 0.010; for internalizing symptoms, *r* = 0.076, 95% CI = [0.03,∞), *t* = 2.44, *P*_one-tailed_ = 0.008; Supplementary Table 6c). Research sites, sex and handedness were included as control variables for the above association analyses and henceforward.

Therefore, we proposed the summed FC strength of positive–positive consensus edges as the NP factor because it was both positively and longitudinally associated with externalizing and internalizing symptoms across adolescence and young adulthood. Notably, the NP factor had a significantly positive FC strength at both ages 14 and 19 (at age 14, *N* = 1,750, *t* = 43.89, Cohen’s *d* = 2.10, 95% CI = [1.98,2.22], *P*_two-tailed_ = 1.36 × 10^−284^; at age 19, *N* = 1,345, *t* = 34.21, Cohen’s *d* = 2.10, 95% CI = [1.74,1.99], *P*_two-tailed_ = 2.53 × 10^−185^), whereas it showed a decreased FC strength from age 14 to age 19 (*N* = 1,087, *t* = 3.12, Cohen’s *d* = 0.19, 95% CI = [0.07,0.31], *P*_two-tailed_ = 0.002). This decrease was associated with baseline behavioral symptoms (*N* = 906; for externalizing symptoms, *r* = 0.15, 95% CI = [0.09,0.21], *t* = 4.68, *P*_two-tailed_ = 3.31 × 10^−6^; for internalizing symptoms, *r* = 0.16, 95% CI = [0.10,0.22], *t* = 4.87, *P*_two-tailed_ = 1.24 × 10^−6^), indicating that individuals with more behavioral symptoms had a distinct NP factor trajectory during this developmental period.

### Prefrontal-related NP factor linked to executive dysfunction

We then characterized the NP factor in its neuroanatomical interpretation (that is, how the NP factor relates to established brain networks and critical brain regions), neurobehavioral relevance (how the NP factor associates with task performance during the MID task and the SST) and associations with common environmental and behavioral risk factors (how the NP factor relates to common putative psychopathological risk factors).

**Fig. 4:**
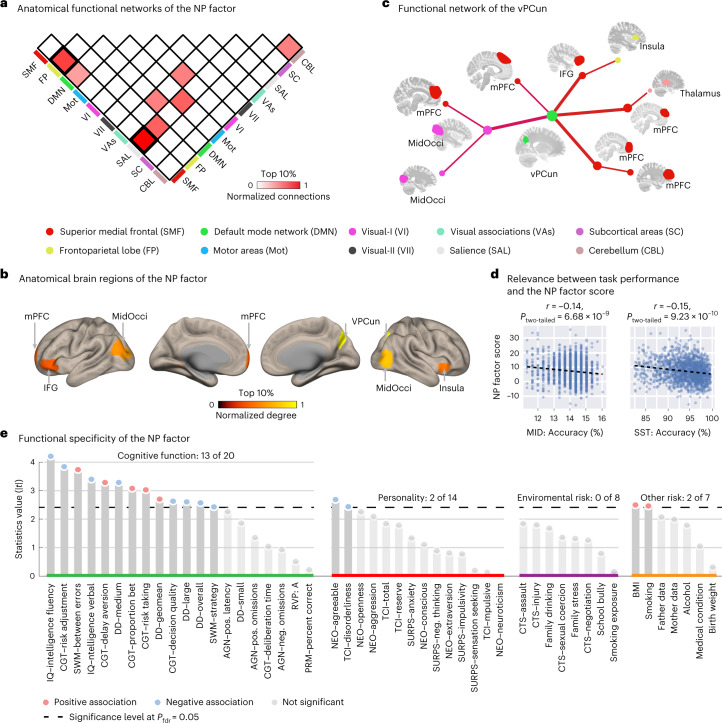
Neurobiological characterization of the NP factor. **a**, The functional brain connections of the NP factor were mainly localized between the frontoparietal network and the superior medial frontal and limbic networks. The color bar indicates the strength of normalized inter- or intranetwork connections, where the number of connections between or within networks was divided by the largest connection number observed. **b**, The top 10% nodes in the NP factor ranked by the normalized node degree (that is, the number of connections with other nodes). **c**, The functional connection network of the NP factor containing the node with the largest degree (that is, the ventral precuneus). **d**, The NP factor was associated with response accuracy during the MID task and the SST. **e**, The NP factor was associated with most cognitive functions (13 of 20), primarily executive function-related behaviors. The significance level (that is, the dashed line) was given as a false discovery rate (fdr) of 0.05. The *P* values were reported as the original value and could survive the multiple correction with Benjamin–Hochberg procedure. |*t*| stands for the absolute value of *t*-statistics. AGN, Affective Go-No Go; BMI, body mass index; DD, Delay Discounting Task; MidOcci, middle occipital cortex; MidPFC, middle prefrontal cortex; NEO, NEO Personality Inventory; RVP: A, Target Sensitivity from Rapid Visual Information Processing task; PRM, Pattern Recognition Memory task; SURPS, Substance Use Risk Personality Scale; SWM, Spatial Working Memory task; TCI, Temperament and Character Inventory–Revised.

The NP factor mainly encompassed prefrontal cortical circuits, such as the superior medial frontal, salience and frontoparietal networks (Fig. 4a and Supplementary Fig. 6), with prominent regions including the ventral precuneus, the inferior frontal gyrus, the middle occipital gyrus, the insula and the medial prefrontal cortex (Fig. 4b and Supplementary Table 8). Notably, the region with the largest node degree (that is, the region with the greatest number of connections to other nodes) was the ventral precuneus, which might serve as a hub that integrates information to or from multiple prefrontal regions^25^ (Fig. 4c).

Because the NP factor was constructed with the SST and the MID task (but not the emotional face task (EFT) due to its failed stability test), we further assessed the associations of the NP factor with task performance during reward sensitivity and inhibitory control. We found that a stronger NP factor was associated with lower accuracy in the MID task (*N* = 1,620, *r* = −0.14, 95% CI = [−0.19,−0.09], *t* = −5.83, *P*_two-tailed_ = 6.68 × 10^−9^) and the SST go trials (*N* = 1,567, *r* = −0.15, 95% CI = [−0.20,−0.10], *t* = −6.16, *P*_two-tailed_ = 9.23 × 10^−10^; Fig. 4d), but not with the reaction time of the MID task (*r* = −0.028, 95% CI = [−0.08,0.02], *t* = −1.11, *P*_two-tailed_ = 0.26) or the stop-signal delay task (*r* = 0.007, 95% CI = [−0.04,0.05], *t* = 0.29, *P*_two-tailed_ = 0.77). A follow-up analysis revealed that the observed differentiated associations with accuracy and reaction time were significant for the NP factor scores derived from both the MID task (*Z* = 3.16, Cohen’s *d* = 0.16, 95% CI = [0.06,0.25], *P*_two-tailed_ = 0.001, Steiger’s *Z*-test) and SST (*Z* = 4.04, Cohen’s *d* = 0.20, 95% CI = [0.11,0.30], *P*_two-tailed_ = 5.35 × 10^−5^, Steiger’s *Z*-test).

We then characterized the functional specificity of the NP factor by systemically investigating its associations with common neurocognitive (that is, cognitive functions and personality) and environmental risk factors for mental disorders. We found the NP factor was predominantly correlated with cognitive functions, such as IQ, risk adjustment and working memory performance (Fig. 4e and Supplementary Table 9), mainly associated with executive control processes^26,27^. In conclusion, our results suggested the NP factor could be the manifestation of deficits in executive control across externalizing and internalizing symptoms.

### NP factor as the endophenotype of comorbid mental disorders

**Fig. 5:**
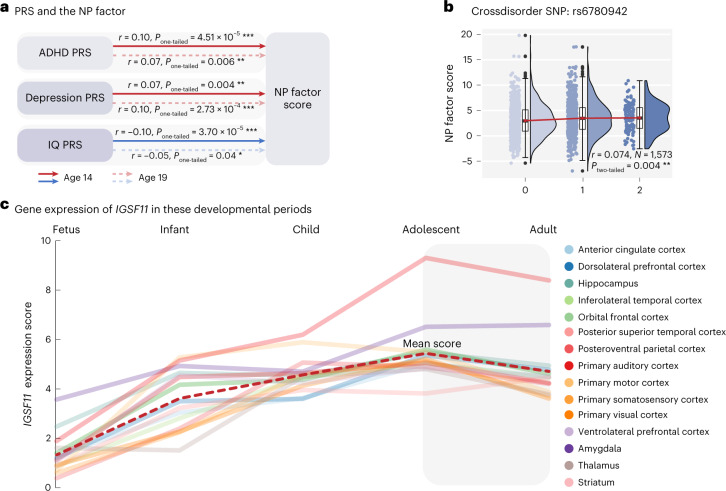
Genetic analyses of the NP factor. **a**, The NP factor correlated with the PRSs of ADHD, depression and IQ, and all were associated with most behavioral symptoms (*N* = 1,594 for age 14; *N* = 1,200 for age 19). **b**, The NP factor was associated with a crossdisorder SNP rs6780942, which was identified in a previous crossdisorder GWAS^18^. This SNP is mapped to the *IGSF11* gene. The upper and lower bars represent the Q3 + 1.5 × IQR and Q1 − 1.5 × IQR, respectively. The upper and lower edges of a box represent the Q3 and Q1, and the central line represents the median. Outliers are illustrated as bold dots. **c**, Expression of *IGSF11* across 15 brain regions peaks at adolescence. The *P* values were reported as the original value and could survive the multiple correction with Benjamin–Hochberg procedure. * *P* < 0.05, ** *P* < 0.01, *** *P* < 0.001. Q1, first quartile; Q3, third quartile; IQR, interquartile range.

We also investigated whether the NP factor could serve as an endophenotype of psychiatric comorbidity using the polygenic risk scores (PRSs) and transdiagnostic genetic variants. We found the NP factor score (that is, the summed FC strength of crossdisorder edges) was associated with the PRS of ADHD^28^, major depressive disorder (MDD)^29^ and IQ^30^, all of which correlated with most behavioral symptoms at age 14 (Supplementary Table 10). Specifically, individuals with a higher NP factor had consistently higher PRSs for both ADHD and MDD, and lower PRSs of IQ at both ages 14 (*N* = 1,594; for ADHD, *r* = 0.10, 95% CI = [0.06,∞), *t* = 3.92, *P*_one-tailed_ = 4.51 × 10^−5^; for MDD, *r* = 0.07, 95% CI = [0.03,∞), *t* = 2.70, *P*_one-tailed_ = 0.004; for IQ, *r* = −0.10, 95% CI = (−∞,−0.06], *t* = −3.97, *P*_one-tailed_ = 3.70 × 10^−5^) and 19 (*N* = 1,200; for ADHD, *r* = 0.070, 95% CI = [0.02,∞), *t* = 2.54, *P*_one-tailed_ = 0.006; for MDD, *r* = 0.10, 95% CI = [0.05,∞), *t* = 3.47, *P*_one-tailed_ = 2.73 × 10^−4^; for IQ, *r* = −0.05, 95% CI = (−∞,−0.002], *t* = −1.80, *P*_one-tailed_ = 0.036; Fig. 5a).

We next investigated candidate biological mechanisms underlying the NP factor by analyzing four single nucleotide polymorphisms (SNPs) identified in a recent large-scale, crossdisorder genome-wide association study (GWAS)^18^. We found that the NP factor was positively associated with the risk allele T of rs6780942 (*N* = 1,573, *r* = 0.074, 95% CI = [0.03,∞), *t* = 3.04, *P*_two-tailed_ = 0.004; Fig. 5b), which was also the most prominent finding in the crossdisorder GWAS^18^ (*P* = 1.11 × 10^−10^) and was significant in both an ADHD^28^ (*P* = 0.0003) and MDD^29^ (*P* = 0.0001) GWAS. The SNP rs6780942 maps to *immunoglobulin superfamily member 11* (*IGSF11*), a gene preferentially expressed in the brain that regulates synaptic adhesion^31^. We then investigated gene expression of *IGSF11* across different developmental periods using BrainSpan^32^ and observed a reduced gene expression of *IGSF11* from adolescence to adulthood (Fig. 5c), which may aid in explaining the reduced connectivity strength of the NP factor during the same period. In summary, these results implied the NP factor might be related to genetically determined neurodevelopment from adolescence to adulthood.

### Generalization of the NP factor

We reconstructed the NP factor on the basis of the MID task and the SST in another large-scale, population-based ABCD study^33^. Again, the NP factor grounded on the ABCD study (*N* = 1,799) showed significant positive associations with both externalizing (*r* = 0.048, 95% CI = [0.001,∞), *t* = 2.04, *P*_one-tailed_ = 0.020) and internalizing symptoms (*r* = 0.056, 95% CI = [0.017,∞), *t* = 2.38, *P*_one-tailed_ = 0.009) at age 10. Moreover, the NP factor estimated at age 10 also demonstrated longitudinal persistence in predicting future behavioral symptoms at age 11 (*N* = 1,042; for externalizing symptoms, *r* = 0.053, 95% CI = [0.002,∞), *t* = 1.72, *P*_one-tailed_ = 0.043; for internalizing symptoms, *r* = 0.079, 95% CI = [0.028,∞), *t* = 2.55, *P*_one-tailed_ = 0.005). This effect was also true for each individual fMRI task (Table 1).

**Table 1:** Generalization of the NP factor in multiple developmental periods across fMRI states for population-based datasets (ABCD, IMAGEN and HCP, *N* = 3,958)

Dataset	Developmental period	Age, mean (s.d.) (yr)	*N* (*n* female, %)	fMRI states or tasks	Externalizing		Internalizing	
					*r*	*P*_one-tailed_	*r*	*P*_one-tailed_
ABCD	Preadolescence	10.0 (0.6)	1,799 (885, 49.2%)	MID, SST	0.048	0.020*	0.056	0.009**
			1,946 (959, 49.3%)	MID	0.040	0.038*	0.047	0.018*
			1,799 (885, 49.2%)	SST	0.042	0.037*	0.042	0.037*
		11.0 (0.7)	1,042 (500, 48.0%)	MID, SST	0.053	0.043*	0.079	0.005**
			1,145 (551, 48.1%)	MID	0.055	0.033*	0.063	0.015*
			1,042 (500, 48.0%)	SST	0.032	0.147	0.056	0.036*
IMAGEN	Young adulthood	18.9 (0.7)	931 (481, 51.7%)	RS	0.063	0.014*		
HCP	Adulthood	28.7 (3.7)	1,081 (605, 56.0%)	RS	0.075	0.007**

**Table 2:** Generalization of the NP factor in multiple developmental periods across fMRI states for clinical case–control datasets (STRATIFY/ESTRA and ADHD-200, *N* = 953)

Dataset	Developmental period	Age, mean (s.d.) (yr)		*N* (*n* female,%)			fMRI states/tasks	Case–control comparison		
		Case	Control	Case	Control			*t*	Cohen’s *d*	*P*_one-tailed_
STRATIFY/ESTRA–all	Young adulthood	23.2 (2.2)	23.5 (1.9)	369 (281, 77.2%)	64 (47, 73.4%)		SST	4.50	0.61	4.43 × 10^−6^***
ESTRA–AN		22.5 (2.1)	23.6 (1.9)	55 (55, 100%)	64 (47, 73.4%)		SST	2.47	0.45	0.007**
ESTRA–BN		22.6 (1.7)	23.6 (1.9)	44 (44, 100%)	64 (47, 73.4%)	SST		2.51	0.49	0.007**
Stratify–AUD		23.3 (2.2)	23.6 (1.9)	127 (74, 58.3%)	64 (47, 73.4%)		SST	4.53	0.69	5.22 × 10^−6^***
Stratify–MDD		23.8 (2.3)	23.6 (1.9)	143 (108, 74.1%)	64 (47, 73.4%)		SST	4.19	0.63	2.07 × 10^−5^***
ADHD-200	Preadolescence	11.0 (2.5)	11.1 (2.4)	228 (50, 21.9%)	292 (145, 50.0%)		RS	3.40	0.30	7.25 × 10^−4^***

For population-based studies ABCD, IMAGEN and HCP, (Table 1) shows the results of the associations of the NP factor scores with externalizing and internalizing symptoms in each cohort. Associations between symptom severities and NP factor scores were estimated using Pearson correlation analysis. For the clinical case–control studies ADHD-200 and STRATIFY/ESTRA (Table 2) shows the group difference in the NP factor scores between the case and control groups. STRATIFY/ESTRA–all includes all cases of anorexia nervosa, bulimia nervosa, alcohol use disorder and MDD. Because higher symptom scores and clinical diagnoses were expected to have higher NP factor scores in these generalization analyses, the corresponding *P* values were calculated using one-tailed tests. Group comparisons of NP factor scores between the clinical case and healthy control groups were conducted using a two-sample *t*-test. The fMRI states/tasks were used to construct the NP factor. The same control sample was used for all diagnoses in STRATIFY/ESTRA.

AN, anorexia nervosa; AUD, alcohol use disorder; BN, bulimia nervosa; MDD, major depression disorder; RS, resting-state fMRI.

* *P* < 0.05, ** *P* < 0.01, *** *P* < 0.001.

**Extended Data Fig. 2:**
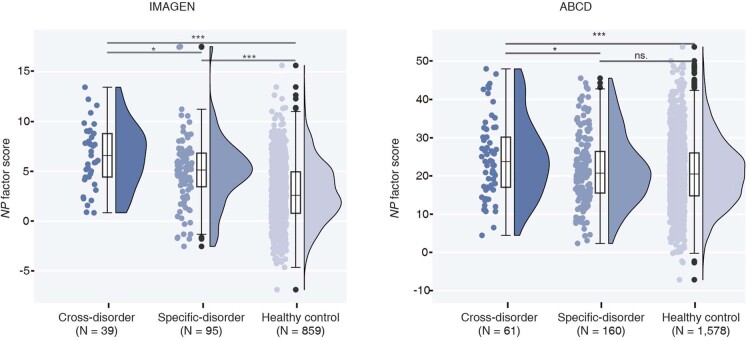
The group difference of the NP factor scores between comorbid-diagnoses, single-diagnosis and healthy control groups. The upper and lower bars represent the Q3 + 1.5xIQR and Q1–1.5xIQR, respectively. Abbreviation: Q1: the 1st quartile; Q3: the 3rd quartile; IQR: the interquartile range; * P < 0.05, ** P < 0.01, *** P < 0.001; ns. not significant.

To assess the clinical relevance of the NP factor, we stratified IMAGEN participants at age 14 into individuals with comorbid diagnoses (that is, those identified as being at severe or high risk for at least two mental disorders simultaneously; *N* = 39), individuals with a single diagnosis (that is, those identified as being at severe or high risk for only one mental disorder; *N* = 95) and healthy controls (that is, those identified as having no mental disorders; *N* = 859; Extended Data Table 1 and Methods). Both those identified as having comorbid diagnoses and those with a single diagnosis demonstrated significantly higher NP factor scores than healthy controls (for comorbid diagnoses, *t* = 7.48, Cohen’s *d* = 1.22, 95% CI = [0.95,∞), *P*_one-tailed_ = 1.80 × 10^−13^; for a single diagnosis, *t* = 6.49, Cohen’s *d* = 0.70, 95% CI = [0.91,∞), *P*_one-tailed_ = 1.39 × 10^−10^). Furthermore, those with comorbid diagnoses also demonstrated significantly higher NP factor scores than those with a single diagnosis (*t* = 2.39, Cohen’s *d* = 0.46, 95% CI = [0.14,∞), *P*_one-tailed_ = 0.018; Extended Data Fig. 2a). Similarly, in the ABCD cohort, using the Kiddie Schedule for Affective Disorders and Schizophrenia–5, we identified 61 individuals with comorbid diagnoses, 160 with a single diagnosis and 1578 healthy controls with no symptoms across all mental disorders (Extended Data Table 2). Again, individuals with comorbid diagnoses demonstrated higher NP factor scores than both those with a single diagnosis (*t* = 2.11, Cohen’s *d* = 0.32, 95% CI = [0.07,∞), *P*_one-tailed_ = 0.017) and healthy controls (*t* = 3.67, Cohen’s *d* = 0.48, 95% CI = [0.26,∞), *P*_one-tailed_ = 2.50 × 10^−4^) (Extended Data Fig. 2b). However, no difference in NP factor scores was observed between those with a single diagnosis and healthy controls (*t* = 1.06, Cohen’s *d* = 0.09, 95% CI = [−0.05,∞), *P*_one-tailed_ = 0.64).

Furthermore, in the case–control cohort STRATIFY/ESTRA (aged 23 years)^34^, the NP factor reconstructed from the SST was significantly higher in individuals with any psychiatric diagnoses (*N* = 369) than in healthy controls (*N* = 64; *t* = 4.50, Cohen’s *d* = 0.61, 95% CI = [0.39,∞), *P*_one-tailed_ = 4.43 × 10^−6^) and for each diagnosis alone (for anorexia nervosa, *N* = 55, *t* = 2.47, Cohen’s *d* = 0.45, 95% CI = [0.15,∞), *P*_one-tailed_ = 0.007; for alcohol abuse, *N* = 127, *t* = 4.53, Cohen’s *d* = 0.69, 95% CI = [0.44,∞), *P*_one-tailed_ = 5.22 × 10^−6^; for bulimia nervosa, *N* = 44, *t* = 2.51, Cohen’s *d* = 0.49, 95% CI = [0.17,∞), *P*_one-tailed_ = 0.007; for MDD, *N* = 143, *t* = 4.19, Cohen’s *d* = 0.63, 95% CI = [0.38,∞), *P*_one-tailed_ = 2.07 × 10^−5^; the same control sample *N*_control_ = 64 was used for all diagnoses; Table 2). Notably, no significant result was observed for the MID task, which might be due to the gradual disassociation between the NP factor scores generated from the SST (inhibitory control) and the MID task (reward sensitivity) with brain maturation (for IMAGEN at age 14, *N* = 1,750, *r* = 0.32, 95% CI = [0.28,0.36], *t* = 14.12, *P*_two-tailed_ = 5.85 × 10^−43^; for STRATIFY/ESTRA at age 23, *N* = 305, *r* = 0.03, 95% CI = [−0.08,0.14], *t* = 0.52, *P*_two-tailed_ = 0.70; *r*_difference_ = 0.29, *Z* = 5.04, *P*_two-tailed_ = 4.65 × 10^−7^).

Finally, we explored whether the NP factor identified during reward processing and inhibitory control could be generalized to predict symptoms on the basis of a highly related NP factor derived from the same FC using resting-state fMRI (*N* = 1,002, *r* = 0.26, 95% CI = [0.20,0.31], *t* = 8.51, *P*_two-tailed_ = 2.22 × 10^−16^), that is, the most abundant fMRI data that are widely available for most population-based and clinical neuroimaging data, and considered as a nonspecific proxy of task-based FC^35^. In the IMAGEN dataset, the NP factor established with resting-state connectivity showed a significant association with externalizing symptoms at age 19 (*N* = 931, *r* = 0.063, 95% CI = [0.01,∞), *t* = 1.91, *P*_one-tailed_ = 0.014; Table 1). Additionally, for healthy adults from the population-based HCP dataset (average age of 29 years), the resting-state NP factor was significantly associated with externalizing symptoms (*N* = 1,081, *r* = 0.075, 95% CI = [0.03,∞), *t* = 2.47, *P*_one-tailed_ = 0.007; Table 1). This association was further validated in the clinical ADHD-200 dataset, which showed significantly higher NP factor scores in individuals with ADHD (*N* = 292) compared with those in the control group (aged 11 years, *N* = 228, *t* = 3.40, Cohen’s *d* = 0.30, 95% CI = [0.15,∞), *P*_one-tailed_ = 7.25 × 10^−4^; Table 2).

## Discussion

In this study, using a large longitudinal neuroimaging genetic cohort, we identified a reliable neural endophenotype (that is, the NP factor) of behavioral symptoms for multiple mental disorders, with implications for early prevention and therapeutics in psychiatry.

We constructed the crossdisorder brain signature through the intersection of externalizing and internalizing edges, rather than identifying the neural correlates associated with the behaviorally defined general p factor^36^, which was recently criticized for its oversimplification^8^. In other words, we assumed that the neural substrates underlying general psychopathology were homogeneously associated with all psychiatric symptoms. Indeed, we also identified a large quantity of dissensus crossdisorder edges between externalizing and internalizing symptoms. However, despite their large quantity, these dissensus edges did not explain more variance than the consensus edges (that is, the NP factor) at age 14 (Fig. 2c). Furthermore, unlike the consensus edges, the dissensus edges lost most of their behavioral associations at age 19 (Fig. 2c), which could explain the surge of comorbid externalizing and internalizing disorders since late adolescence^37^, that is, when the consensus edges or the NP factor begin to dominate the associations with behavioral measures.

The transdiagnostic NP factor mainly targeted top-down regulatory prefrontal circuits, such as the frontoparietal, superior medial frontal and salience networks. This is in line with previous findings that altered activations or gray matter volume in these cognitive networks may have further implications in emotional and reward and punishment processing, which leads to the wide range of psychiatric symptoms seen with both fMRI and structural MRI studies^38,39^. However, whereas previous research overwhelmingly identified reduced transdiagnostic neural substrates (that is, hypoactivation, hypoconnectivity and decreased gray matter volume; Supplementary Table 11), the NP factor identified in this study manifested with hyperconnectivity of prefrontal-related neural circuits underlying general psychopathology. The hyperconnectivity (or hyperactivation) of prefrontal circuits in psychiatric disorders is usually explained as a neural compensation of executive resources to the less efficient integration of bottom-up sensory information^9,39,40^. However, Cabeza et al.^41^ argued that, when associated with cognitive deficits, hyperfunctioning prefrontal circuits might not necessarily represent a protective compensation effect but rather a disruption of the efficacy of executive control.

Indeed, hyperconnectivity of the NP factor may result from delayed brain development. During adolescence, the brain undergoes the maturational processes of synaptic pruning and synapse stabilization^42^ to improve the efficiency of information transmission in the brain, leading to gradually reduced gray matter volume in the healthy brain over time. However, such a reduction (from ages 14 to 19) was significantly inhibited in individuals with a higher NP factor score (*N* = 1,132, *r* = −0.176, 95% CI = [−0.23,−0.12], *t* = −6.01, *P*_two-tailed_ = 2.50 × 10^−9^), which indicates atypical trajectories of neural circuit maturation in individuals with high NP factor scores. Furthermore, compared with the somatosensory and motor cortices, the synaptic elimination process in the frontal and parietal lobes is delayed and prolonged during adolescence. Therefore, both brain regions might be more vulnerable to maldevelopment^42^, which is consistent with our observation that the NP factor is enriched in the frontal and parietal lobes. Finally, individuals with higher NP factor scores at baseline and follow-up showed increased behavioral symptoms and widespread deficits of cognitive control, which is a function long associated with prefrontal and parietal cortices.

Remarkably, we found the NP factor is associated with *IGSF11*, a gene implicated in the neuronal adhesion molecule that binds to and stabilizes AMPA receptors regulating synapse stabilization^31^. The upregulation of synaptic adhesion molecules prevents the process of synaptic pruning^43^, which is the signature morphological event of late brain maturation during adolescence^44^. Expression of *IGSF11* decreases from adolescence to adulthood, which might mediate the developmental trajectory of the NP factor during this period. Therefore, genetic evidence convincingly suggests the proposed NP factor represents an endophenotype of prefrontal delayed development across externalizing and internalizing symptoms.

The NP factor identified in adolescents was generalizable across multiple developmental periods and showed consistent prediction of multiple behavioral symptoms (such as ADHD, CD, anxiety and depression) in population-based data of preadolescents (ABCD, aged 10–11 years), adolescents (IMAGEN, aged 14 years) and young adults (IMAGEN, aged 19 years) and clinical data of young adults (STRATIFY/ESTRA, aged 23 years). Many psychiatric disorders emerge during the transition from adolescence to adulthood^45–47^, that is, the period in which the brain undergoes its final phase of maturation^48^. Therefore, the NP factor identified during this critical period may mark the fast-evolving and most vulnerable neural network from preteenager to adult^45^, thus revealing the neuropsychopathological mechanisms underlying the behavioral symptoms related to psychiatric disorders, before onset of clinical illness^49^.

Nevertheless, more rigorous experimental studies are needed to clarify the causal mechanisms underlying this NP factor. Additionally, although we focused on a general neuropsychopathology in this study, factors of other more specific forms of psychopathology (such as externalizing, internalizing and thought disorder psychopatholgoies) should also play essential roles. Therefore, future studies are required to elucidate the dynamic interaction between the general and specific neuropsychopathologies that may further contribute to the development of psychiatric comorbidity. It should also be noted that, although the primary nonclinical, large-scale, population-based datasets used in this study were designed to represent the broader population (that is, no exclusion criterion was set for mental health status) from preadolescence to early adulthood (a critical development period in which the onset of most psychiatric disorders peaks^4,5^), the recruitment of these studies may still suffer from certain sampling biases (for instance, the IMAGEN sample was primarily recruited from middle-class schools and might underrepresent participants with the most severe psychiatric symptoms^50^). Therefore, despite the large sample sizes in our population-based data (IMAGEN and ABCD), these data may have lower base rates of psychiatric disorders, especially for extreme cases, than in the broader population. Therefore, future studies need to have longitudinal data from the same participants, with sufficient representations of the most severe symptoms, to verify whether the NP factor behaves in a dimensional manner (that is, either quantitatively or qualitatively differentiated between clinical participants and healthy controls) and could be extended to other developmental periods, such as middle and late adulthood.

In conclusion, we established a transdiagnostic NP factor that could be generalized to multiple large-scale, population-based and clinical neuroimaging datasets and is persistent from preadolescence to early adulthood. The NP factor could bridge the genetic substrates of neurodevelopmental processes and higher-order cognitive deficits. These results demonstrated that the NP factor could serve as a reliable neuropsychopathological biomarker of psychiatric comorbidity, substantially advancing our knowledge in stratified psychiatric medicine.

## Methods

### Study protocol

We investigated the multivariate associations between behavioral symptoms and task-based FC (MID task, SST and emotion reactivity task) with the widely used CPM^23,51^. The task-based connectome prediction analysis was conducted in the population-based IMAGEN sample of children aged 14 years. Additional analyses were then performed to discover the relationships between behavioral symptoms and crossdisorder neural circuits. Next, the predictive and crossdisorder connectome was investigated at several levels, using behavioral, longitudinal, genetic and clinical data. Notably, because psychiatric comorbidity is common in both males and females, we mainly focused on identifying the crossdisorder neural circuits across the whole population, not specifically for each sex.

### IMAGEN

IMAGEN is a large-scale longitudinal neuroimaging–genetics cohort study (*N* = 2,000 at age 14, *N* = 1,300 at age 19) conducted to understand the biological basis of individual variability in psychological and behavioral traits and their relationship to common psychiatric disorders. The study involves a thorough neuropsychological, behavioral, clinical and environmental assessment of each participant. Participants also undergo biological characterization with the collection of T1-weighted structural MRI, task-based fMRI and genetic data. In this investigation, we used task and resting-state MRI, genetic and behavioral data. Notably, as a population-based approach, IMAGEN has balanced sample sizes for male and female participants (based on self-reported sex).

### Development and Well-Being Assessment and Strengths and Difficulties Questionnaire

Behavioral symptoms of the IMAGEN participants were assessed using screening questions from the Development and Well-Being Assessment (DAWBA)^52^ and the Strengths and Difficulties Questionnaire (SDQ)^53^. DAWBA is a wide-ranging psychiatric screening questionnaire that was previously used to define subthreshold clinical symptoms in neuroimaging studies of subclinical psychopathology^54^. The SDQ was also used in this investigation because it contributes to the assignment of diagnostic status in the DAWBA^52^. At age 14, the parent-rated externalizing symptoms comprised ADHD (23 items), ODD (11 items), CD (10 items) and ASD (7 items). The child-rated internalizing symptoms included GAD (7 items), depression (8 items), SP (13 items) and ED (5 items). The full set of psychiatric questions asked in our investigation can be found in Supplementary Table 1. The choice of using different versions of questionnaires (that is parent-rated externalizing symptoms and child-rated internalizing symptoms) at age 14 was grounded on findings that externalizing problem scores from parents are more reliable than those from children themselves, and vice versa^55^. At age 19, however, because parent-rated questionnaires were unavailable, we used child-rated questionnaires for both externalizing and internalizing symptoms (Supplementary Table 1).

DAWBA also provides a diagnostic output for common psychiatric disorders, that is, the likelihood of a clinical diagnosis being made after rating. Of the 1,750 IMAGEN participants at age 14, 134 had a high risk for at least one diagnosis (that is, they scored 4 or 5, with over 50% chance of being diagnosed), and 39 participants met the criteria for two or more diagnoses. More specifically, 93 participants were likely to have one or more externalizing disorders (24 with ADHD, 45 with ODD, 59 with CD and 1 with ASD), and 46 participants were likely to have one or more internalizing disorders (16 with GAD, 21 with depression, 5 with ED and 14 with SP; see Extended Data Table 1 for more detail).

### Monetary incentive delay task

Participants performed a modified version of the MID task (Supplementary Fig. 1) to examine neural responses to reward anticipation and reward outcome^56^. The task consisted of 66 10-second trials. In each trial, participants were presented with one of three cue shapes (cue, 250 ms) denoting whether a target (white square) would subsequently appear on the left or right side of the screen and whether zero, two or ten points could be won in that trial. After a variable delay (4,000–4,500 ms) of fixation on a white crosshair, participants were instructed to respond with a left or right button press as soon as the target appeared. Feedback on whether any, and how many, points were won during the trial was presented for 1,450 ms after the response (Supplementary Fig. 1). With a tracking algorithm, task difficulty (that is, target duration varied between 100 and 300 ms) was individually adjusted, such that each participant successfully responded on ~66% of trials. Participants had first completed a practice session outside the scanner (~5 minutes) during which they were instructed that, for every five points won, they would receive one food snack in the form of small chocolate candies. Our study used the task conditions consisting of hit anticipation, hit feedback and miss feedback.

### Stop-signal task

Participants performed an event-related SST (Supplementary Fig. 2) designed to study neural responses to successful and unsuccessful inhibitory control^57^. The task comprised go trials and stop trials. During go trials (83%, 480 trials), participants were presented with arrows pointing either to the left or to the right. Participants were then instructed to make a button response with their left or right index finger, corresponding to the direction of the arrow. In the unpredictable stop trials (17%, 80 trials), the arrows pointing left or right were followed (on average 300 ms later) by arrows pointing upwards; participants were instructed to inhibit their motor responses during these trials. A tracking algorithm changes the time interval between the go and stop signal onsets according to each participant’s performance on previous trials (average percentage of inhibition over previous stop trials, recalculated after each stop trial), resulting in 50% successful and 50% unsuccessful inhibition trials. The intertrial interval was 1,800 ms. The tracking algorithm of the task ensured that participants were successful on 50% of stop trials and worked at the edge of their own inhibitory capacity. Our study used the SST measures consisting of stop success, stop failure and go wrong.

### Emotional face task

The EFT was adapted from Grosbras et al.^58^. Participants watched 18-second blocks of either a face movie (depicting anger or neutrality) or a control stimulus. Each face movie showed black and white video clips (200–500 ms) of male or female faces. Five blocks each of angry and neutral expressions were interleaved with nine blocks of the control stimulus. Each block contained eight trials of six face identities (three female). The same identities were used for the angry and neutral blocks. The control stimuli were black and white concentric circles that expanded and contracted at various speeds, roughly matching the contrast and motion characteristics of the face clips. Our study used the EFT task conditions of neutral and angry faces.

### Image acquisition

fMRI data were acquired at eight IMAGEN assessment sites with 3 T MRI scanners from different manufacturers (Siemens, Philips, General Electric, Bruker). The scanning variables were specifically chosen to be compatible with all scanners. The same scanning protocol was used at all sites. In brief, high-resolution T1-weighted 3D structural images were acquired for anatomical localization and coregistration with the functional time series. In addition, blood oxygen level-dependent (BOLD) functional images were acquired with gradient-echo, echo-planar imaging sequence. For all tasks, each volume consisted of 40 slices aligned to the anterior commission–posterior commission line (2.4-mm slice thickness, 1-mm gap). The echo time was optimized (30 ms, with repetition time (TR) of 2,200 ms) to provide reliable imaging of the subcortical areas.

### Task-based functional image preprocessing

Task-based fMRI data were first prepreprocessed using SPM8 (Statistical Parametric Mapping, http://www.fil.ion.ucl.ac.uk/spm). Spatial preprocessing included slice time correction to adjust for time differences due to multislice imaging acquisition, realignment to the first volume in line, nonlinearly warping to the MNI space (on the basis of a custom echo-planar imaging template (53 × 63 × 46 voxels) created from an average of the mean images from 400 adolescents), resampling at a resolution of 3 × 3 × 3 mm^3^ and smoothing with an isotropic Gaussian kernel of 5 mm full-width at half-maximum.

### Network construction

To estimate the condition-specific FC, we used the CONN toolbox (version 16.h) with the weighted generalized linear model method. Task condition regressors, 21 covariate regressors (21 covariate regressors consisting of 12 motion regressors (3 translations, 3 rotations and 3 translations shifted 1 TR before, and 3 translations shifted 1 TR later) and 9 additional columns corresponding to the long-term effects of the movement (3 nuisance variables for the white matter and 6 nuisance variables for ventricles, commonly referred to as CompCor correction^59^) were first regressed out from the raw BOLD signal of each region of interest (ROI). The residual signals were then further fed into weighted generalized linear models to investigate conditional time-series correlations (that is, the conditional FC) between any pairs of ROIs, where the temporal weight function for each condition was calculated as the corresponding, but now rectified, task condition regressor (that is, only time points expected with positive BOLD signals count). This approach not only amplifies the expected hemodynamic delay to each task condition but also deweights the initial and final scans when estimating functional correlation measures to avoid spurious jumps in BOLD signal and reduces the potential crosstalk between adjacent task conditions^60^. After this procedure, ROI:ROI FCs were calculated on the basis of the brain template from the 268-node functional brain atlas^22^ (Supplementary Fig. 3).

### Connectome-based predictive modeling

We used CPM (Supplementary Fig. 4) to predict the participants’ behavioral symptoms from whole-brain, task-based FC. CPM is a recently developed method for identifying functional brain connections related to a behavior variable of interest, which is then used to predict behavior in novel participants (that is, participants whose data were not used in model creation)^23^. The CPM procedure was recently described in studies reporting its application to cognitive and psychiatry variables, such as fluid intelligence, attention control and ADHD^51,61–63^. The CPM processing pipeline is available online (https://www.nitrc.org/projects/bioimagesuite/). We slightly modified the original CPM, which used the leave-one-out crossvalidation, to a 50-fold crossvalidation process to hasten the process while maintaining robustness. In the first step, we randomly divided the data into 50 folds, where one fold was left out as the testing dataset while the other 49 folds were used as the training dataset. Next, a vector of behavioral scores (for example, ADHD symptoms) was associated with the edge of the connectome (that is, the FC matrix) across participants from the training dataset, with site and handedness being included as covariates. Then, a default threshold^23^ (that is, *P* < 0.01 in our study) was applied to retain only edges that were significantly associated (either positively or negatively) with behavioral symptoms in the training dataset. Analyses were also repeated with three additional thresholds (for example, 0.05, 0.005 and 0.001), demonstrating similar predictive performance (Supplementary Table 2). Next, the sum of the weights of positive and negative edges (negative edges will be multiplied by −1 before summing up) was calculated for each individual and entered into a linear regression model to estimate the relationship between the summed edge strength and the observed behavior in the training dataset. In the testing dataset, the summed edge strength of each individual was submitted to the corresponding linear model estimated in the training dataset to generate the predicted behavior score. This process was repeated 50 times, with predicted behavior scores in each testing fold established on the basis of the remaining 49-fold data. Finally, Spearman’s correlation was applied to estimate the model performance between predicted and actual behavior scores across all individuals. We repeated the CPM 1,000 times and continued further analyses using the edges selected in over 95% of models to select the most robust edges. For more details on CPM, see Shen et al.^23^.

### Neuropsychopathology factor

The NP factor was constructed to represent longitudinally consistent and generalizable transdiagnostic brain signatures across externalizing and internalizing spectra. First, by applying CPM on condition-specific functional neural networks (that is, the functional connectome derived for each task condition), we identified crossdisorder edges that were associated with at least one externalizing symptom and one internalizing symptom simultaneously. Then, for each task condition, we investigated if the number of crossdisorder edges identified was significantly higher than a random observation using a permutation test (see Reliability assessment using permutation tests for more details). Only the significant, and therefore informative, task conditions and their crossdisorder edges were retained for further analyses. Next, given that different combinations of association directions with externalizing and internalizing symptoms have distinct neurobiological implications, we stratified these crossdisorder edges into four groups to improve interpretability: positive–positive (or negative–negative) edges that were associated with both externalizing and internalizing symptoms positively (or negatively); positive–negative edges that were associated positively with externalizing symptoms but negatively with internalizing symptoms; and negative–positive edges of negative associations with externalizing symptoms but positive associations with internalizing symptoms. Lastly, the four groups of crossdisorder edges were investigated for longitudinal consistency on the basis of their predictive performance on both externalizing and internalizing symptoms in the follow-up study at age 19, and the longitudinally consistent crossdisorder edges (that is, the FC strength) were summed to generate the NP factor. Please note that only positive–positive edges (that is, edges positively associated with both internalizing and externalizing symptoms) were found to be longitudinally consistent and used to compute the NP factor. Therefore, the NP factor may serve as a transdiagnostic neural indicator for comorbid externalizing and internalizing symptoms.

### Reliability assessment using permutation tests

To investigate which task conditions provided reliable crossdisorder edges, we implemented permutation tests evaluating if identified crossdisorder edges from each task condition were indeed informative, that is, if the number of edges identified for the given condition was significantly larger than that in a random discovery (Supplementary Fig. 5). Due to the time-consuming nature of the proposed CPM analysis (1,000 repetitions of 50-fold crossvalidation as described in Connectome-based predictive modeling), the number of permutations was set as 1,000, which was sufficient to provide an accurate estimation of a *P* value as small as 0.01. This permutation process was also used to provide unbiased *P* values for the association of the crossdisorder network with behavioral symptoms.

### Generalization datasets

To investigate whether the NP factor identified with the adolescent IMAGEN dataset using the task-based connectomes could be generalized into other developmental periods and fMRI states, we used multiple, large-scale, population-based datasets (ABCD cohort^33^ and the HCP^64^) and clinical case–control datasets (STRATIFY/ESTRA^34^ and ADHD-200^65^).

#### ABCD cohort

The dataset used for this study was selected from the Annual Curated Data Release (https://data-archive.nimh.nih.gov/abcd) of the ABCD cohort, which recruited 11,875 children between 9 and 11 years of age from 21 sites across the USA^66^. MRI data in the ABCD study were collected from different 3 T scanner platforms (Siemens Prisma, General Electric MR750 and Philips Achieva dStream). To minimize the biases introduced by multiple platforms, we only included MRI data from the most frequent manufacturer, Siemens Prisma; data from this manufacturer comprised 5,968 participants from 13 sites. By examining the similarity of brain activations across these 13 sites, we further selected 2,326 participants with consistent activation patterns from 4 sites. After quality control^67^, 1,966 participants of the MID task and 1,837 participants of the SST were included in further analysis. ABCD has balanced sample sizes for boys and girls (based on self-reported sex) (Table 1). To construct the NP factor in the ABCD dataset, with the same positive–positive edges used to establish the NP factor in the IMAGEN cohort, we extracted the corresponding FC of reward anticipation and reward positive feedback from the MID task and FC of the stop success and stop failure from the SST. The sum of FCs for the MID task and SST was the corresponding NP factor for the ABCD. For psychiatric symptoms, we used the Parent Child Behavior Checklist Scores (abcd_cbcls01) to assess the dimensional psychopathology in children^68^. The summed scores of externalizing and internalizing symptoms were used in further analysis. The ABCD Parent Diagnostic Interview for Diagnostic and Statistical Manual of Mental Disorders, Fifth Edition (DSM-5) provides a diagnostic output for common psychiatric disorders (abcd_ksad01). Diagnosis of ASD was provided from a clinical assessment questionnaire (abcd_screen01). Because the morbidity of SP (21.5%) with abcd_ksad01 in the ABCD dataset was much higher than that of other pediatric epidemiologic investigations of SP (4.8%)^69,70^, we excluded this diagnostic information in the clinical relevance analysis. For all analyses of ABCD data, we included site, family, handedness and sex as covariates in a mixed model^71^.

#### HCP

The dataset used for this investigation was selected from the March 2017 public data release from the HCP, WU-Minn Consortium. HCP has balanced sample sizes for men and women (based on self-reported sex; Table 1). Our sample included 1,081 participants (aged 22–35 years, mean age 31 years) scanned on a 3 T Siemens connectome-Skyra scanner. More details of participants and collection and preprocessing of data are provided at the HCP website (http://www.humanconnectome.org/). Externalizing symptoms were measured using the Achenbach Adult Self-Report (ASR) Syndrome Scales^72^ (ASR_Computed_Externalizing_Adjusted_T). For all analyses of HCP data, we included site, handedness and sex as covariates.

#### Stratify and ESTRA

STRATIFY and ESTRA recruited participants (ages 19–25) with alcohol use disorder or major depression (STRATIFY), anorexia nervosa or bulimia nervosa (ESTRA), and controls with no mental disorder diagnosis at three sites (Berlin, London and Southampton). The proportions of men and women (based on self-reported sex) varied across different mental health disorder groups (Table 1). Furthermore, the protocol of both studies was harmonized to match the IMAGEN protocol. These datasets collected task-based neuroimaging data of the SST and MID task. After quality control (the same quality control procedures as with the ABCD dataset^67^), 267 cases and 46 controls of the MID task and 380 cases and 64 controls of the SST were included in further analysis. For all analyses of Stratify and ESTRA data, we included site, handedness and sex as covariates.

#### ADHD-200

ADHD-200 is a grassroots initiative dedicated to accelerating the scientific community’s understanding of the neural basis of ADHD (aged 7–21 years). Males are predominant in the case group whereas both sexes (based on self-reported sex) are balanced in the control group (Table 1). Inclusion criteria included no history of neurological diseases and other chronic medical conditions and estimates of full-scale IQ above 80, and psychostimulant drugs were withheld at least 24–48 hours before scanning. Data were downloaded from the ADHD-200 consortium website (http://fcon_1000.projects.nitrc.org/indi/adhd200). In our study, we used data from four sites (Peking University, Kennedy Krieger Institute, New York University Child Study Center and Oregon Health & Science University) that recruited both participants with ADHD and control participants without ADHD. In total, there were 228 cases and 292 controls. For all analyses of ADHD-200 data, we included site, handedness and sex as covariates.

### Genotyping for the IMAGEN study

DNA purification and genotyping were performed by the Centre National de Génotypage. DNA was extracted from whole-blood samples (∼10 ml) preserved in BD Vacutainer EDTA Tubes (Becton, Dickinson and Company) using the Gentra Puregene Blood Kit (QIAGEN), according to the manufacturer’s instructions. SNPs with call rates of <98%, minor allele frequency <1% or deviation from the Hardy–Weinberg equilibrium (*P* < 1.00 × 10^−4^) were excluded from analyses. Individuals with an ambiguous sex code, excessive missing genotypes (failure rate >2%) and outlying heterozygosity (heterozygosity rate of 3 s.d. from the mean) were also excluded.

### Polygenic risk scores

To calculate the PRSs of depression, ADHD and intelligence, we used previously published GWASs of ADHD^28^, depression^29^ and intelligence^30^. The discovery depression GWAS consisted of 135,458 cases and 344,901 controls, the ADHD study consisted of 20,183 cases and 35191 controls and the IQ study included 269,867 individuals. We then used PRSice software (http://prsice.info/) to calculate the corresponding PRS. The clumping process was applied to retain only SNPs with the smallest *P* value for each linkage disequilibrium block (combined with a sliding window process to exclude any less significant SNPs with an *r*^2^ < 0.1 in 250-kb windows). PRSs were calculated at *P* value thresholds between 0 and 0.5 in increments of 0.01, and we used the mean PRSs of depression, ADHD and intelligence for subsequent analyses^73^.

### Cognition–behavior phenotypes

#### Cambridge Cognition Battery

The Cambridge Cognition Battery (http://www.cambridgecognition.com/) comprised the Spatial Working Memory task (number of errors and strategies), the Cambridge Guessing Task (CGT; risk taking, quality of decision-making, delay aversion, deliberation time, overall proportion bet, risk adjustment), the Rapid Visual Information Processing task and the Affective Go-No Go task (mean correct latency for positive and negative stimuli, number of omission errors for positive and negative stimuli). The CGT quality of decision-making is the proportion of trials on which the participant chooses the most likely outcome. The CGT deliberation time is the reaction time to choose the color of the box. The overall bet is the overall bet across the trials. CGT risk taking is mean proportion of available points the participant stakes at each trial. CGT delay aversion is the difference between the risk-taking score in the descending and the ascending conditions. CGT risk adjustment is the degree to which a participant adjusts their risk taking according to the ratio of colored boxes, calculated as [2 × (proportion of points staked (%) at 9:1) + (% 8:2) − (% 7:3) − 2 × (% 6:4)] ÷ CGT risk taking. The Rapid Visual Information Processing task is a 10-minute test that measures sustained attention by presenting a rapid stream of digits and requiring participants to detect target sequences. A white box is displayed in the center of the screen, in which digits 2–9 are rapidly presented at 100 digits per minute. Participants are required to detect target sequences (for example, 2-4-7, 3-5-7 or 4-6-8) and respond to this target sequence as quickly as possible. Outcome measures include a signal detection theory measure of target sensitivity and mean response latency.

#### IQ

We measured intelligence using the fluency and verbal components of the Wechsler Intelligence Scale for Children, Fourth Edition^74^.

#### Delay discounting

We used the Monetary-Choice Questionnaire, as described by Kirby^75^. The Monetary-Choice Questionnaire is an efficient and reliable measurement of delay discounting that has been validated in adolescents^76^. For each participant, we estimated the *k* values that reflect how one discounts a reward value with the delay required to obtain it. The questionnaire contains 27 dichotomous-choice items pitting a smaller immediate reward against a larger delayed reward for three levels of reward magnitude (small, medium and large). Higher *k* coefficients in a hyperbolic discounting equation for each reward level represent greater preference for small immediate rewards and higher impulsivity. The geometric mean was calculated and logarithmically transformed to use in our analyses.

### Personality

#### Substance Use Risk Personality Scale

The Substance Use Risk Personality Scale (23 items, self-questionnaire) was used to measure sensation seeking, impulsivity, anxiety sensitivity and negative thinking subscores, and has been shown to be related to substance use in adolescents^77^.

#### NEO Personality Inventory

The NEO Personality Inventory (60 items, self-questionnaire) explores the big-five domains of personality: neuroticism, extraversion, openness, agreeableness and conscientiousness^78^.

#### Temperament and Character Inventory–Revised

The Temperament and Character Inventory–Revised (36 items)^79^ was used to measure excitability, impulsiveness, reserve, disorderliness and their combined measure of novelty seeking.

### Substance use

#### Alcohol

Alcohol abuse was assessed using the screening questions from the Alcohol Use Disorders Identification Test (AUDIT, ten items)^80^. The AUDIT was developed by the World Health Organization as a simple way to screen and identify people who are at risk of developing alcohol problems. AUDIT focuses on identifying the preliminary signs of hazardous drinking and mild dependence. It is used to detect alcohol problems experienced within the last year, and it is one of the most accurate alcohol screening tests available.

#### Smoking

Smoking behavior was assessed as the frequency (that is, cigarettes per day) of smoking during the last 30 days using the European School Survey Project on Alcohol and Other Drugs^81^.

### Environmental risk

#### Childhood Trauma Questionnaire

The Childhood Trauma Questionnaire (CTQ)^82^ was used to assess childhood maltreatment across childhood and adolescence. It consists of five domains: emotional abuse, emotional neglect, physical abuse, physical neglect and sexual abuse. The scores from the five domains was summed for a total CTQ score; the higher the score the greater the severity of maltreatment.

#### School bully

School bully behavior was measured using an adapted questionnaire grounded on the Health Behaviour in School-aged Children survey. These questions were initially used in the revised Olweus Bully/Victim Questionnaire^83^.

#### Family stress

Family stress was measured using the family stress and socioeconomic item from the DAWBA. A larger score for this item indicates greater family stress.

#### Family drinking

Family drinking was measured using the parent AUDIT.

### Other risks

#### Body mass index

Recorded weight and height were used to calculate the body mass index (weight in kilograms per height in meters squared).

#### Pregnancy and Birth Questionnaire

The Pregnancy and Birth Questionnaire was used to collect information during the pregnancy; it consisted of mother and father data, medical condition of mother (‘did the mother take any prescribed medication during pregnancy?’), smoking exposure (‘how many cigarettes did the mother smoke per day before pregnancy?’) and birth weight (‘what was the birth weight of the child?’).

### Ethical approval

The IMAGEN study was approved by local ethics research committees at each research site: King’s College London, University of Nottingham, Trinity College Dublin, University of Heidelberg, Technische Universität Dresden, Commissariatà l’Energie Atomique et aux Energies Alternatives and University Medical Center. Informed consent was sought from all participants and a parent/guardian of each participant. The ABCD study conforms to each site’s institutional review board’s rules and procedures, and all participants provided informed consent (parents) or informed assent (children). The WU-Minn HCP Consortium obtained full informed consent from all participants, and research procedures and ethical guidelines were followed in accordance with the institutional review boards. ADHD-200 is a multicenter study, and each site was approved by the local research ethics review board. Signed informed consent was obtained from all participants or their legal guardians before participation. STRATIFY/ESTRA was approved by the London – Westminster Research Ethics Committee, and signed informed consent was obtained from all participants. Compensation for time and travel costs were provided for participants in the above cohorts, as approved by the ethical committees.

### Reporting summary

Further information on research design is available in the Nature Portfolio Reporting Summary linked to this article.

## Online content

Any methods, additional references, Nature Portfolio reporting summaries, source data, extended data, supplementary information, acknowledgements, peer review information; details of author contributions and competing interests; and statements of data and code availability are available at 10.1038/s41591-023-02317-4.

### Supplementary information

Supplementary Information Supplementary Figs. 1–6.

Reporting Summary

Supplementary Tables Supplementary Tables 1–11.

## Figures and Tables

**Extended Data Table 1:**
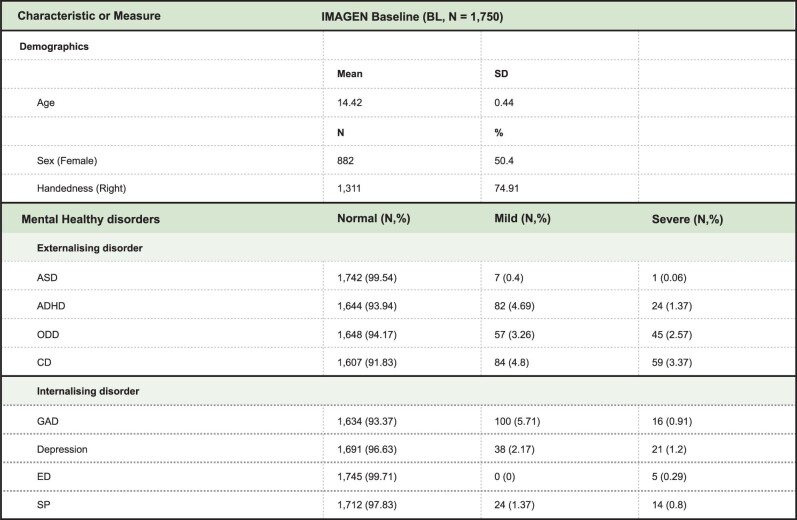
Characteristics of the IMAGEN cohort at age 14

The development and well-being assessment (DAWBA) provides a diagnostic output for common psychiatric disorders with six ranks from 0 to 5. Specifically, for each mental disorder, individuals were flagged as normal if scored 0 or 1 (that is less than 0.1% and approximately 0.5% of children have the disorder in question, respectively), as low risk (that is with mild symptoms) if scored 2 or 3 (approximate 3% or 15% of children have the disorder in question, respectively), and as high risk (that is with severe symptoms) if scored 4 or 5 (approximate 50% or more than 70% of children have the disorder in question, respectively). ADHD, attention-deficit/hyperactivity disorder; ASD, autism spectrum disorder; CD, conduct disorder; ODD, oppositional defiant disorder; GAD, general anxiety disorder; ED, eating disorder; SP, specific phobia.

**Extended Data Table. 2:**
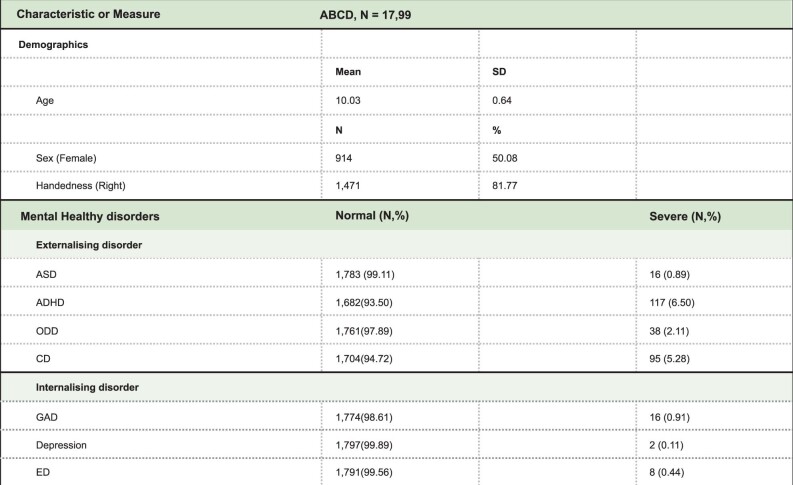
Characteristics of the ABCD cohort at age 10

The ABCD Parent Diagnostic Interview for DSM-5 Full provides a diagnostic output for common psychiatric disorders (abcd_ksad01). The diagnosis of ASD was provided in a clinical assessment questionnaire (abcd_screen01). ADHD, attention-deficit/hyperactivity disorder; ASD, autism spectrum disorder; CD, conduct disorder; ODD, oppositional defiant disorder; GAD, general anxiety disorder; ED, eating disorder. Please note that as the morbidity of specific phobia (SP) (21.5%) with abcd_ksad01 in the ABCD dataset was much higher than that of other pediatric epidemiologic investigations of SP (4.8%), we thus excluded this diagnosis here.

## Data Availability

IMAGEN data are available from a dedicated database at https://imagen2.cea.fr. STRATIFY/ESTRA data are available from the IMAGEN database at https://imagen2.cea.fr. ABCD data are available from a dedicated database at https://abcdstudy.org/. HCP data are available from a dedicated database at https://www.humanconnectome.org/. ADHD-200 data are available from a dedicated database at http://fcon_1000.projects.nitrc.org/indi/adhd200. Shen 268 parcellation is available at https://www.nitrc.org/frs/?group_id=51.

## Code availability

The code that supports the findings of this study is available on GitHub at https://github.com/xic199wzr/NP-factor.
