# Author Correction: A shared neural basis underlying psychiatric comorbidity

**DOI:** 10.1038/s41591-023-02512-3

**Published:** 2023-08-09

**Authors:** Chao Xie, Shitong Xiang, Chun Shen, Xuerui Peng, Jujiao Kang, Yuzhu Li, Wei Cheng, Shiqi He, Marina Bobou, M. John Broulidakis, Betteke Maria van Noort, Zuo Zhang, Lauren Robinson, Nilakshi Vaidya, Jeanne Winterer, Yuning Zhang, Sinead King, Tobias Banaschewski, Gareth J. Barker, Arun L. W. Bokde, Uli Bromberg, Christian Büchel, Herta Flor, Antoine Grigis, Hugh Garavan, Penny Gowland, Andreas Heinz, Bernd Ittermann, Hervé Lemaître, Jean-Luc Martinot, Marie-Laure Paillère Martinot, Frauke Nees, Dimitri Papadopoulos Orfanos, Tomáš Paus, Luise Poustka, Juliane H. Fröhner, Ulrike Schmidt, Julia Sinclair, Michael N. Smolka, Argyris Stringaris, Henrik Walter, Robert Whelan, Sylvane Desrivières, Barbara J. Sahakian, Trevor W. Robbins, Gunter Schumann, Tianye Jia, Jianfeng Feng, IMAGEN Consortium, Tobias Banaschewski, Gareth J. Barker, Arun L. W. Bokde, Uli Bromberg, Christian Büchel, Herta Flor, Antoine Grigis, Hugh Garavan, Penny Gowland, Andreas Heinz, Bernd Ittermann, Jean-Luc Martinot, Marie-Laure Paillère Martinot, Frauke Nees, Dimitri Papadopoulos Orfanos, Tomáš Paus, Luise Poustka, Juliane H. Fröhner, Michael N. Smolka, Henrik Walter, Robert Whelan, Sylvane Desrivières, Tianye Jia, Gunter Schumann, STRATIFY/ESTRA Consortium, Marina Bobou, M. John Broulidakis, Zuo Zhang, Lauren Robinson, Nilakshi Vaidya, Jeanne Winterer, Yuning Zhang, Sinead King, Gareth J. Barker, Arun L. W. Bokde, Hervé Lemaître, Frauke Nees, Dimitri Papadopoulos Orfanos, Ulrike Schmidt, Julia Sinclair, Argyris Stringaris, Henrik Walter, Robert Whelan, Sylvane Desrivières, Gunter Schumann, Betteke Maria van Noort, ZIB Consortium, Chao Xie, Shitong Xiang, Wei Cheng, Gunter Schumann, Tianye Jia, Jianfeng Feng

**Affiliations:** 1 Institute of Science and Technology for Brain-Inspired Intelligence, Fudan University, Shanghai, China; 2 Key Laboratory of Computational Neuroscience and Brain-Inspired Intelligence (Fudan University), Ministry of Education, Shanghai, China; 3 Faculty of Psychology, Technische Universität Dresden, Dresden, Germany; 4 School of Health Sciences, The University of Manchester, Manchester, UK; 5 Social Genetic and Developmental Psychiatry Centre, Institute of Psychiatry, Psychology and Neuroscience, King’s College London, London, UK; 6 Clinical and Experimental Sciences, Faculty of Medicine, University of Southampton, Southampton, UK; 7 Department of Psychology, College of Science, Northeastern University, Boston, MA USA; 8 Department of Psychology, MSB Medical School Berlin, Berlin, Germany; 9 Department of Psychological Medicine, Section for Eating Disorders, Institute of Psychiatry, Psychology and Neuroscience, King’s College London, London, UK; 10 South London and Maudsley NHS Foundation Trust, London, UK; 11 Centre for Population Neuroscience and Stratified Medicine (PONS), Department of Psychiatry and Neuroscience, Charité Universitätsmedizin Berlin, Berlin, Germany; 12 Department of Psychiatry and Psychotherapy, Charité – Universitätsmedizin Berlin, corporate member of Freie Universität Berlin, Humboldt-Universität zu Berlin, and Berlin Institute of Health, Berlin, Germany; 13 Department of Education and Psychology, Freie Universität Berlin, Berlin, Germany; 14 Psychology Department, B44 University Rd, University of Southampton, Southampton, UK; 15 School of Medicine, Center for Neuroimaging, Cognition and Genomics, National University of Ireland (NUI), Galway, Ireland; 16 Department of Child and Adolescent Psychiatry and Psychotherapy, Central Institute of Mental Health, Medical Faculty Mannheim, Heidelberg University, Mannheim, Germany; 17 Department of Neuroimaging, Institute of Psychiatry, Psychology and Neuroscience, King’s College London, London, UK; 18 Discipline of Psychiatry, School of Medicine and Trinity College Institute of Neuroscience, Trinity College Dublin, Dublin, Ireland; 19 University Medical Centre Hamburg-Eppendorf, Hamburg, Germany; 20 Institute of Cognitive and Clinical Neuroscience, Central Institute of Mental Health, Medical Faculty Mannheim, Heidelberg University, Mannheim, Germany; 21 Department of Psychology, School of Social Sciences, University of Mannheim, Mannheim, Germany; 22 NeuroSpin, C.E.A., Université Paris-Saclay, Gif-sur-Yvette, France; 23 Departments of Psychiatry and Psychology, University of Vermont, Burlington, VT USA; 24 Sir Peter Mansfield Imaging Centre School of Physics and Astronomy, University of Nottingham, Nottingham, UK; 25 Department of Psychiatry and Psychotherapy, Charité–Universitätsmedizin Berlin, corporate member of Freie Universität Berlin, Humboldt-Universität zu Berlin, and Berlin Institute of Health, Berlin, Germany; 26 Physikalisch-Technische Bundesanstalt (PTB), Braunschweig and Berlin, Germany; 27 Institut National de la Santé et de la Recherche Médicale, UMR 992 INSERM, CEA, Faculté de médecine, Université Paris-Sud, Université Paris-Saclay, NeuroSpin, Gif-sur-Yvette, France; 28 Institut National de la Santé et de la Recherche Médicale, INSERM U1299 ‘Trajectoires développementales en psychiatrie’, Université Paris-Saclay, Ecole Normale supérieure Paris-Saclay, CNRS UMR9010, Centre Borelli, Gif-sur-Yvette, France; 29 AP-HP, Sorbonne Université, Department of Child and Adolescent Psychiatry, Pitié-Salpêtrière Hospital, Paris, France; 30 Institute of Medical Psychology and Medical Sociology, University Medical Center Schleswig-Holstein, Kiel University, Kiel, Germany; 31 Department of Psychiatry and Neuroscience and Centre Hospitalier Universitaire Sainte-Justine, University of Montreal, Quebec, Canada; 32 Department of Child and Adolescent Psychiatry and Psychotherapy, University Medical Centre Göttingen, Göttingen, Germany; 33 Department of Psychiatry and Neuroimaging Center, Technische Universität Dresden, Dresden, Germany; 34 Division of Psychiatry and Department of Clinical, Educational & Health Psychology, University College London, London, UK; 35 School of Psychology and Global Brain Health Institute, Trinity College Dublin, Dublin, Ireland; 36 Department of Psychology and Behavioural and Clinical Neuroscience Institute, University of Cambridge, Cambridge, UK; 37 Department of Sports and Health Sciences, University of Potsdam, Potsdam, Germany; 38 PONS Centre, Institute for Science and Technology of Brain-inspired Intelligence (ISTBI), Fudan University, Shanghai, China; 39 School of Mathematical Sciences and Centre for Computational Systems Biology, Fudan University, Shanghai, China; 40 Department of Computer Science, University of Warwick, Coventry, UK; 41 Fudan ISTBI–ZJNU Algorithm Centre for Brain-inspired Intelligence, Zhejiang Normal University, Jinhua, China

**Keywords:** Psychiatric disorders, Cognitive control

Correction to: *Nature Medicine* 10.1038/s41591-023-02317-4. Published online 24 April 2023.

In the version of this article initially published, the STRATIFY data also included cohort data from the ESTRA consortium, though this was not acknowledged in the author list and the section in Methods on the Stratify dataset. The Methods are now updated, and the author list is amended to combine the STRATIFY and ESTRA consortium names and to include the following authors: Marina Bobou, M. John Broulidakis, Betteke Maria van Noort, Zuo Zhang, Lauren Robinson, Nilakshi Vaidya, Jeanne Winterer, Yuning Zhang, Sinead King, Hervé Lemaître, Ulrike Schmidt, Julia Sinclair, Argyris Stringaris and Sylvane Desrivières. The STRATIFY and ESTRA consortia are now combined to list Marina Bobou, M. John Broulidakis, Betteke Maria van Noort, Zuo Zhang, Lauren Robinson, Nilakshi Vaidya, Jeanne Winterer, Yuning Zhang, Sinead King, Gareth J. Barker, Arun L. W. Bokde, Hervé Lemaître, Frauke Nees, Dimitri Papadopoulos Orfanos, Ulrike Schmidt, Julia Sinclair, Argyris Stringaris, Henrik Walter, Robert Whelan, Sylvane Desrivières and Gunter Schumann as members, and the IMAGEN consortium is updated to also include Sylvane Desrivières. Affiliations, author contributions and acknowledgements have been updated to reflect the new authorship, and all changes have been made in the HTML and PDF versions of the article.

