# Supplementary material for: A shared neural basis underlying psychiatric comorbidity
Source: Nat Med. 2023 Apr 24;29(5):1232–42. doi: 10.1038/s41591-023-02317-4 (PMC10202801; doi:10.1038/s41591-023-02317-4)
Supplement: Supplementary file 1 — Supplementary Figs. 1–6. [file 41591_2023_2317_MOESM1_ESM.pdf]

nature medicine

Article https://doi.org/10.1038/s41591-023-02317-4

A shared neural basis underlying psychiatric comorbidity

In the format provided by the authors and unedited

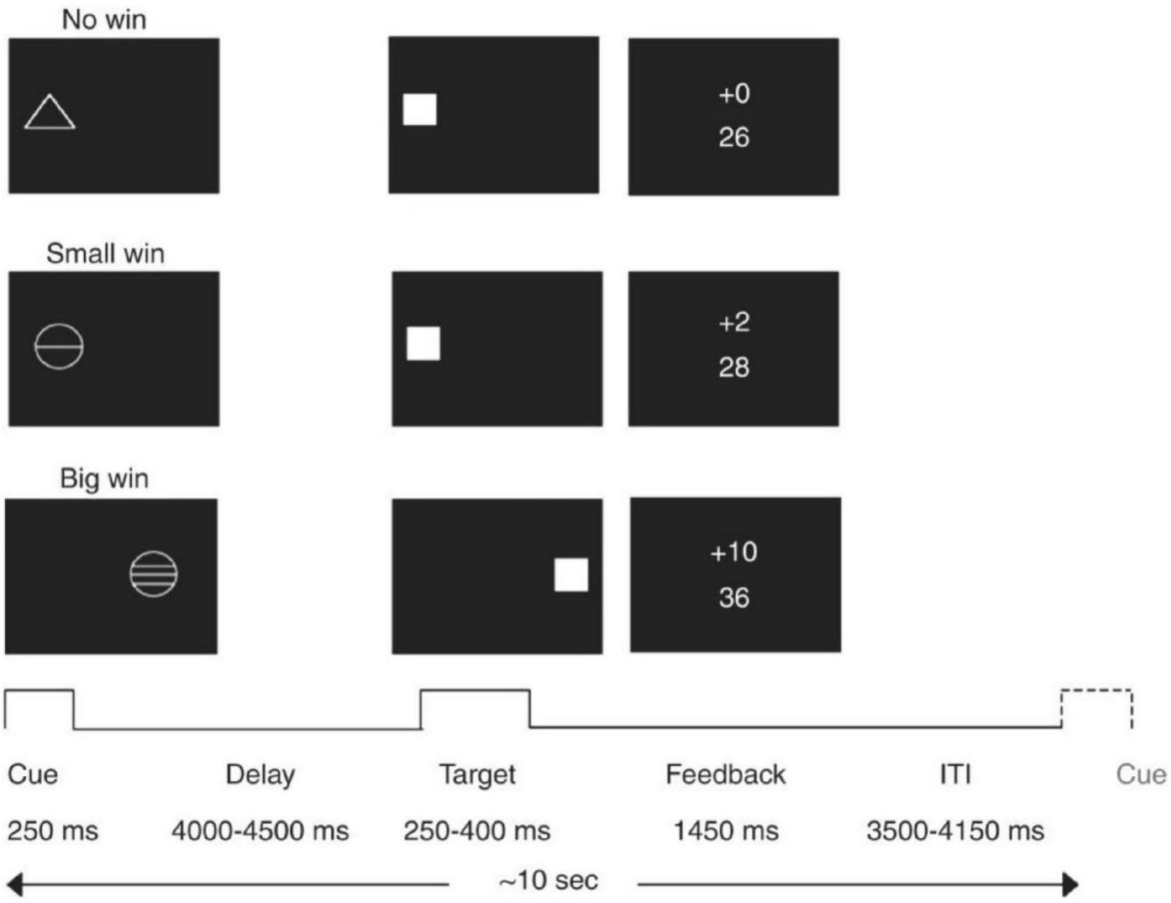

Figure S1. The task design of the Monetary Incentive Delay (MID) Task.

The figure of experimental paradigm was adapted from a previous publication (Jia, T., et al. Neurobehavioural characterisation and stratification of reinforcement-related behaviour. Nat Hum Behav 4, 544-558 (2020).).

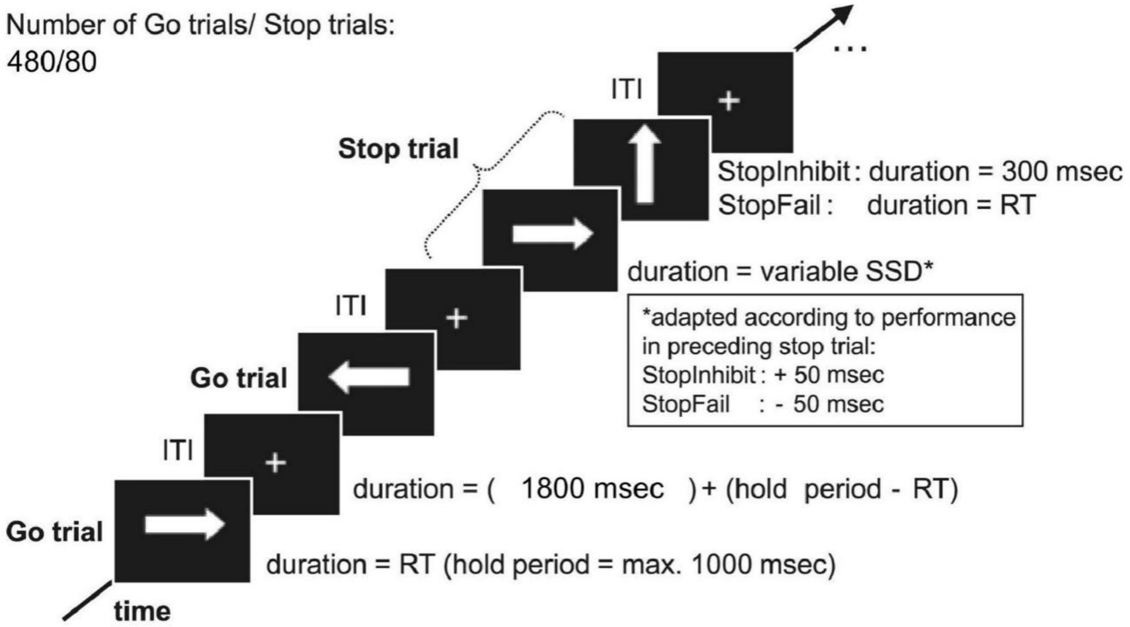

Figure S2. The task design of the stop signal Task (SST).

The figure of experimental paradigm was adapted from a previous publication (Jia, T., et al. Neurobehavioural characterisation and stratification of reinforcement-related behaviour. Nat Hum Behav 4, 544-558 (2020).)..

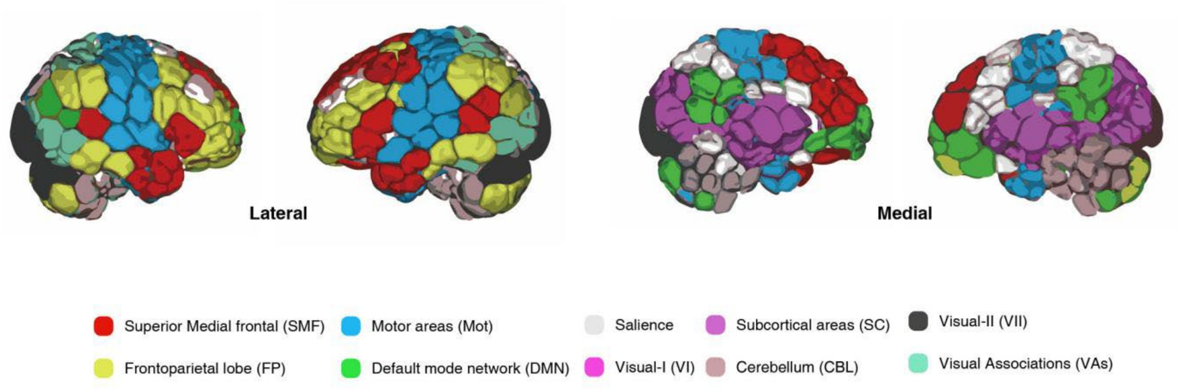

Figure S3. Ten large-scale canonical networks for anatomical interpretation.

The figure of shen-268 template was adapted from a previous publication (Greene, A.S., Gao, S., Scheinost, D. & Constable, R.T. Task-induced brain state manipulation improves prediction of individual traits. Nature communications 9, 2807 (2018)).

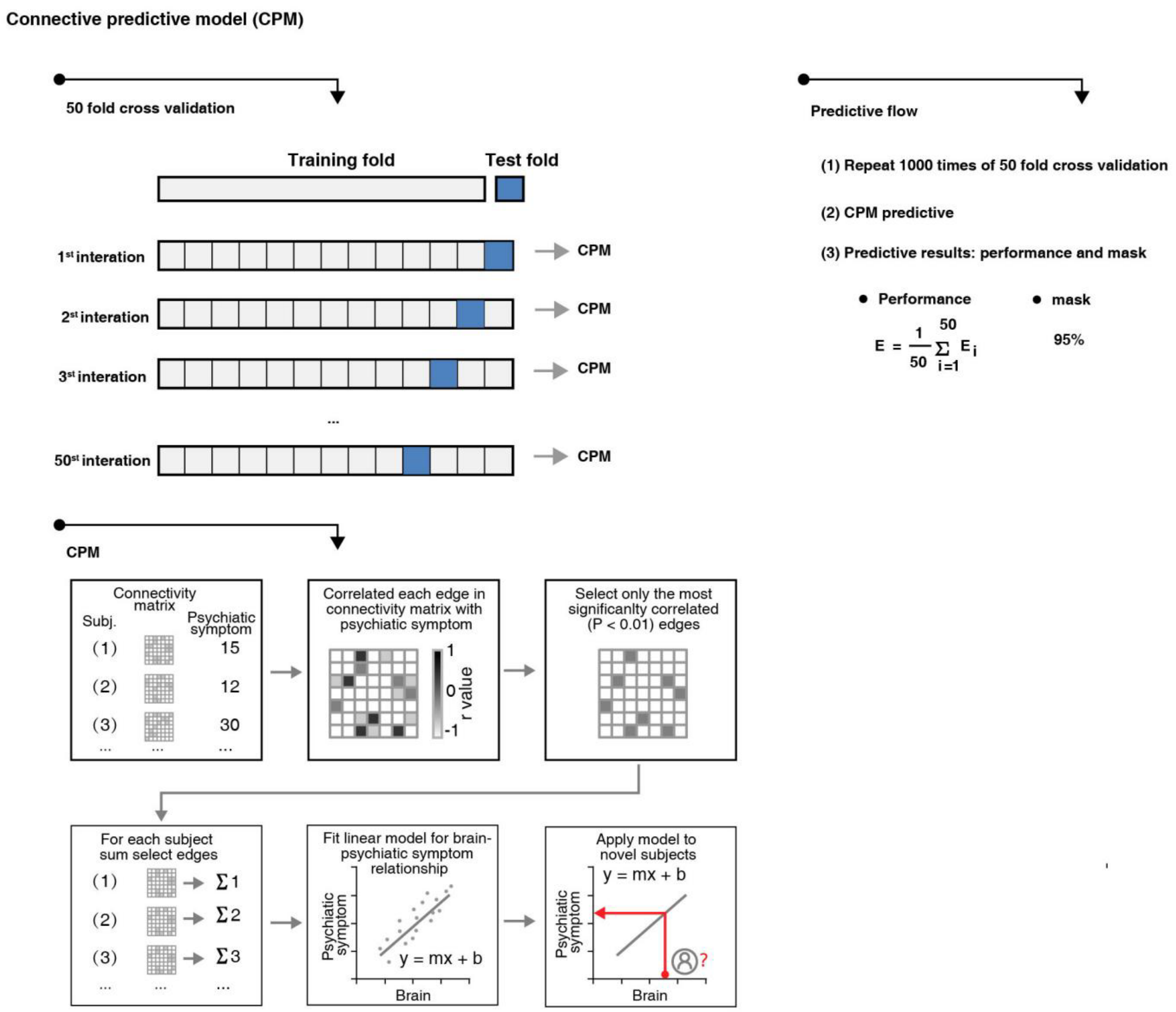

$$E = \frac{1}{50}\sum_{i=1}^{50} E_i$$

Figure S4. The analysis flow of the connectome-based predictive model (CPM).

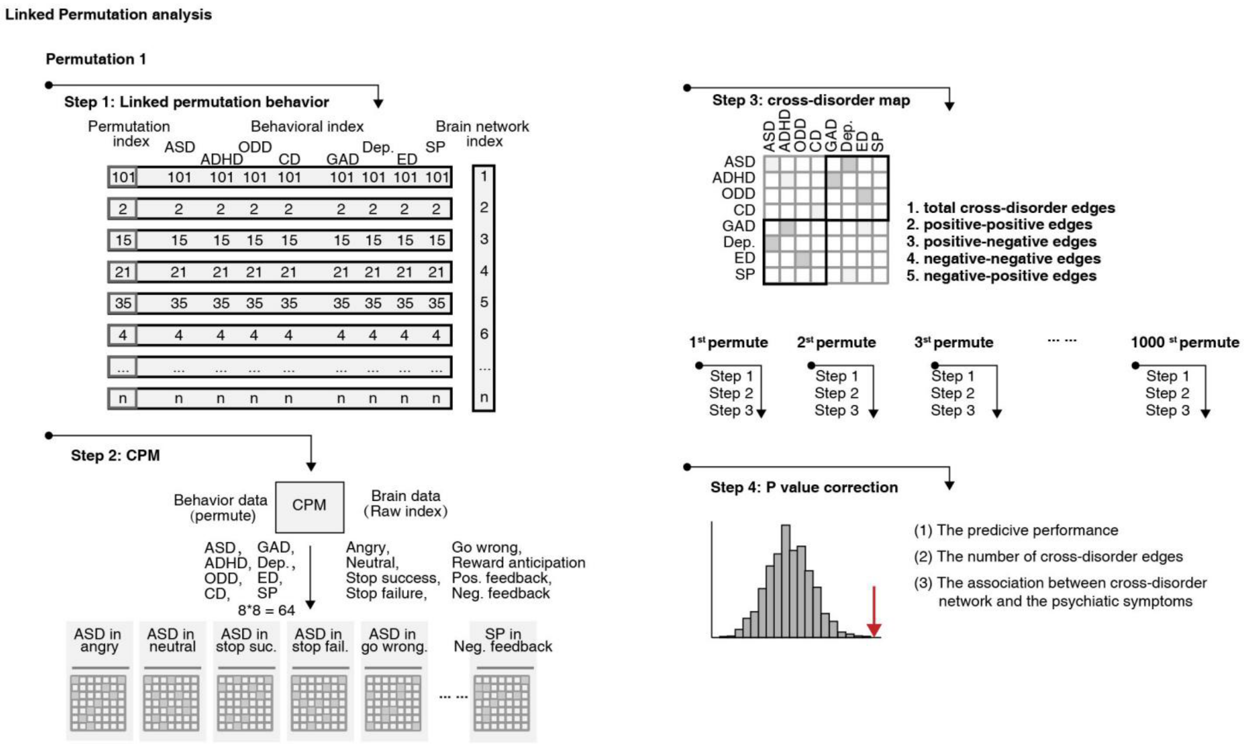

Figure S5. The analysis flow of the permutation analysis.

ADHD, attention deficit/hyperactivity disorder; ASD, autism spectrum disorder; CD, conduct disorder; ODD, oppositional defiant disorder; Pos. Feedback, positive feedback; Neg. Feedback, negative feedback.

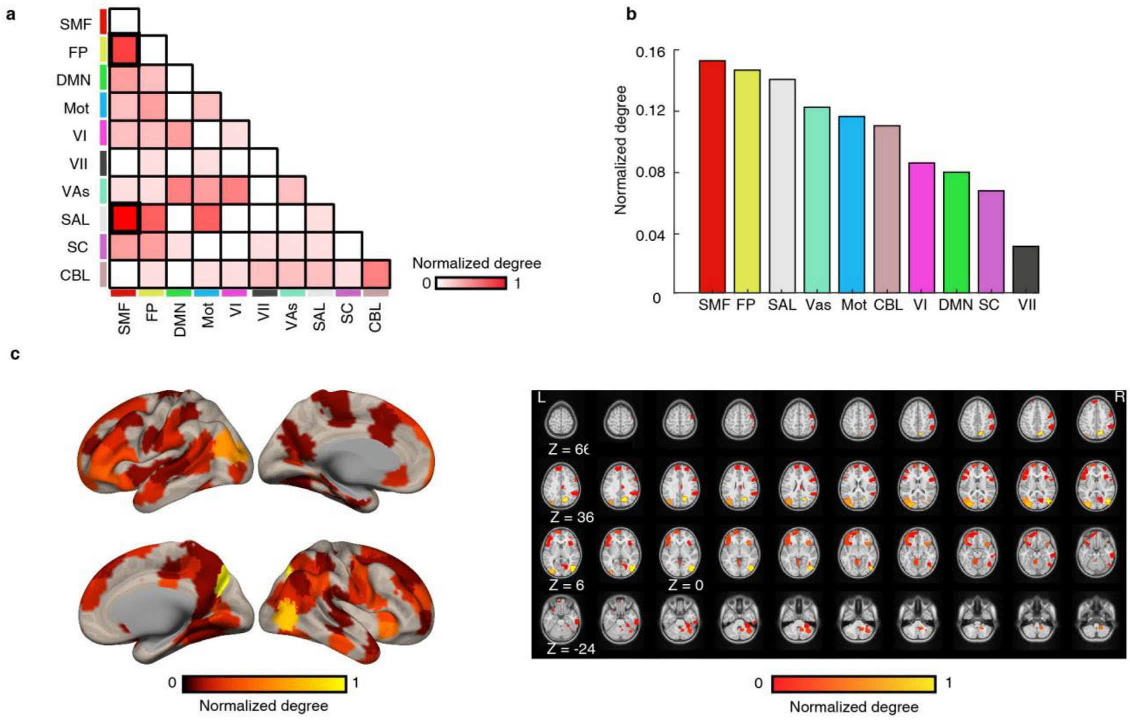

Figure S6. The network-level and regional normalised degrees of the *NP* factor. a**.** The normalised network degrees of the *NP* factor between and within the ten large-scale networks. **b.** The summed normalised network degrees of the *NP* factor for the ten large-scale networks. **c.** The normalised regional degrees of the *NP* factor.
