# Supplementary material for: A shared neural basis underlying psychiatric comorbidity
Source: Nat Med. 2023 Apr 24;29(5):1232–42. doi: 10.1038/s41591-023-02317-4 (PMC10202801; doi:10.1038/s41591-023-02317-4)
Supplement: Supplementary file 2 — Reporting Summary [file 41591_2023_2317_MOESM2_ESM.pdf]

nature portfolio

Corresponding author(s): Tianye Jia

Last updated by author(s): Mar 9, 2023

Reporting Summary

Nature Portfolio wishes to improve the reproducibility of the work that we publish. This form provides structure for consistency and transparency in reporting. For further information on Nature Portfolio policies, see our Editorial Policies and the Editorial Policy Checklist.

Statistics

For all statistical analyses, confirm that the following items are present in the figure legend, table legend, main text, or Methods section.

n/a	Confirmed	
☐	☒	The exact sample size (*n*) for each experimental group/condition, given as a discrete number and unit of measurement
☐	☒	A statement on whether measurements were taken from distinct samples or whether the same sample was measured repeatedly
☐	☒	The statistical test(s) used AND whether they are one- or two-sided<br>*Only common tests should be described solely by name; describe more complex techniques in the Methods section.*
☐	☒	A description of all covariates tested
☐	☒	A description of any assumptions or corrections, such as tests of normality and adjustment for multiple comparisons
☐	☒	A full description of the statistical parameters including central tendency (e.g. means) or other basic estimates (e.g. regression coefficient) AND variation (e.g. standard deviation) or associated estimates of uncertainty (e.g. confidence intervals)
☐	☒	For null hypothesis testing, the test statistic (e.g. *F*, *t*, *r*) with confidence intervals, effect sizes, degrees of freedom and *P* value noted<br>*Give P values as exact values whenever suitable.*
☒	☐	For Bayesian analysis, information on the choice of priors and Markov chain Monte Carlo settings
☐	☒	For hierarchical and complex designs, identification of the appropriate level for tests and full reporting of outcomes
☐	☒	Estimates of effect sizes (e.g. Cohen's *d*, Pearson's *r*), indicating how they were calculated

Our web collection on statistics for biologists contains articles on many of the points above.

Software and code

Policy information about availability of computer code

Data collection	Psytool platform was used to collect data for both SDQ and DAWBA assessment
Data analysis	All statistical analyses were conducted in the Matlab R2018b. Preprocessing and first level analyses of task-based fMRI data were conducted in SPM8 and CONN.16.h.<br><br>Code Availability<br>The code that supports the findings of this study is available on GitHub: https://github.com/xic199wzr/NP-factor.

For manuscripts utilizing custom algorithms or software that are central to the research but not yet described in published literature, software must be made available to editors and reviewers. We strongly encourage code deposition in a community repository (e.g. GitHub). See the Nature Portfolio guidelines for submitting code & software for further information.

Data

Policy information about availability of data

All manuscripts must include a data availability statement. This statement should provide the following information, where applicable:

- Accession codes, unique identifiers, or web links for publicly available datasets
- A description of any restrictions on data availability
- For clinical datasets or third party data, please ensure that the statement adheres to our policy

IMAGEN data are available from a dedicated database: https://imagen2.cea.fr;
Stratify data are also available from the IMAGEN database: https://imagen2.cea.fr;
ABCD data are available from a dedicated database: https://abcdstudy.org/;
HCP data are available from a dedicated database: https://www.humanconnectome.org/;
ADHD-200 data are available from a dedicated database: http://fcon_1000.projects.nitrc.org/indi/adhd200;
Shen 268 parcellation is available from https://www.nitrc.org/frs/?group_id=51.

Data availability Statement
IMAGEN data are available from a dedicated database: https://imagen2.cea.fr; Stratify data are also available from the IMAGEN database: https://imagen2.cea.fr; ABCD data are available from a dedicated database: https://abcdstudy.org/; HCP data are available from a dedicated database: https://www.humanconnectome.org/; ADHD-200 data are available from a dedicated database: http://fcon_1000.projects.nitrc.org/indi/adhd200. Shen 268 parcellation is available from https://www.nitrc.org/frs/?group_id=51

Human research participants

Policy information about studies involving human research participants and Sex and Gender in Research.

Reporting on sex and gender	We used the term sex throughout this manuscript.<br>As psychiatric comorbidity is common in both males and females, we mainly focused on identifying the cross-disorder neural circuits across the whole population but not specific for each sex.<br>As a population-based approach, the IMAGEN cohort has balanced sample sizes for male and female participants (based on self-reported sex; Table1).
Population characteristics	Healthy Caucasian adolescents at age 14 were recruited from middle-class school across Europe. 1750 participants investigated in this study, clinical DAWBA ratings are available. For diagnosis information, out of the 1750 IMAGEN participants at age 14, 134 had high risk for at least one diagnosis (i.e. scored 4 or 5, with over 50% chance of been diagnosed), and 39 participants meets criteria for two or more diagnoses. More specifically, 93 participants were likely to have one or more externalising disorders: 24 with attention-deficit/hyperactivity disorder (ADHD), 45 with oppositional defiant disorder (ODD), 59 with conduct disorder (CD), and 1 with autism spectrum disorder (ASD); and 46 participants were likely to have one or more internalising disorders: 16 with general anxiety disorder (GAD), 21 with depression, 5 with eating disorder (ED), and 14 with specific phobia (SP) .
Recruitment	Healthy Caucasian adolescents at age 14 were recruited from middle-class school from multiple sites across Europe (London, Nottingham, Dublin, Paris, Manhannm, Berlin, Dresden, Humberg).
Ethics oversight	The IMAGEN study was approved by local ethics research committees at each research site: King's College London, University of Nottingham, Trinity College Dublin, University of Heidelberg, Technische Universität Dresden, Commissariat à l'Energie Atomique et aux Energies Alternatives, and University Medical Center. Informed consent was sought from all participants and a parent/guardian of each participant.

Note that full information on the approval of the study protocol must also be provided in the manuscript.

Field-specific reporting

Please select the one below that is the best fit for your research. If you are not sure, read the appropriate sections before making your selection.

☒ Life sciences ☐ Behavioural & social sciences ☐ Ecological, evolutionary & environmental sciences

For a reference copy of the document with all sections, see nature.com/documents/nr-reporting-summary-flat.pdf

Life sciences study design

All studies must disclose on these points even when the disclosure is negative.

Sample size	Over 1700 individuals with complete data of fMRI or behaviours were involved in the present study. This sample size is sufficiently to detect an effect size as little as R^2 = 2% with a statistical power over 95% at the significance level 0.0001.
Data exclusions	Individuals with incomplete data across fMRI and behaviours were excluded in the cross-disorder network analysis.

Replication	The resting-state fMRI of the IMAGEN study was used for internal replication/generalisation; data from the ABCD, HCP,ADHD-200 and Striatify studies were used for external replication/generalisation. The above replication analyses were all conducted independently. The majority of replication analyses were successful except for two analyses that 1) at age 11 in ABCD, the NP factor score generated only with the SST was not significant associated with the externalising symptoms (Table 1); 2) in Stratify, the NP factor score generated only with the MID task does not differentiate between any of the clinical groups and the healthy control group.
Randomization	As a population study, no randomization was conducted. Nevertheless, covariates (e.g. research sites, handedness and sex) were included wherever suitable to control for potential confounding effects.
Blinding	As a population-based observational study, the IMAGEN cohort does not focus on any specific exposures or outcomes and no intervention or treatment was applied. Therefore, blinding was not needed to avoid bias in sampling or experimental processes.

Reporting for specific materials, systems and methods

We require information from authors about some types of materials, experimental systems and methods used in many studies. Here, indicate whether each material, system or method listed is relevant to your study. If you are not sure if a list item applies to your research, read the appropriate section before selecting a response.

Materials & experimental systems

n/a	Involved in the study
☒	☐ Antibodies
☒	☐ Eukaryotic cell lines
☒	☐ Palaeontology and archaeology
☒	☐ Animals and other organisms
☒	☐ Clinical data
☒	☐ Dual use research of concern

Methods

n/a	Involved in the study
☒	☐ ChIP-seq
☒	☐ Flow cytometry
☐	☒ MRI-based neuroimaging

Magnetic resonance imaging

Experimental design

Design type	two even-related tasks: Monetary Incentive Delay Task (MID), Stop Signal Task (SST); one block design task: Emotional Face Task (EFT)
Design specifications	MID: The task consisted of 66 10-second trials. In each trial, participants were presented with one of three cue shapes (cue, 250 ms) denoting whether a target (white square) would subsequently appear on the left or right side of the screen and whether 0, 2 or 10 points could be won in that trial. After a variable delay (4,000-4,500 ms) of fixation on a white crosshair, participants were instructed to respond with left/right button-press as soon as the target appeared. Feedback on whether and how many points were won during the trial was presented for 1,450 ms after the response.<br><br>SST:The task was composed of Go trials and Stop trials. During Go trials (83%; 480 trials) participants were presented with arrows pointing either to the left or to the right. During these trials, subjects were instructed to make a button response with their left or right index finger corresponding to the direction of the arrow. In the unpredictable Stop trials (17%; 80 trials), the arrows pointing left or right were followed (on average 300 ms later) by arrows pointing upwards; participants were instructed to inhibit their motor responses during these trials.<br><br>EFT: Participants watched 18-second blocks of either a face movie (depicting anger or neutrality) or a control stimulus. Each face movie showed black and white video clips (200-500ms) of male or female faces. Five blocks each of angry and neutral expressions were interleaved with nine blocks of the control stimulus. Each block contained eight trials of 6 face identities (3 female). The same identities were used for the angry and neutral blocks. The control stimuli were black and white concentric circles expanding and contracting at various speeds that roughly matched the contrast and motion characteristics of the face clips.
Behavioral performance measures	For both event related tasks MID and SST, performance tracking systems were implemented to adjust difficulty of the tasks to ensure the overall performance of each participant (i.e. successfully responded on ~66% of trials in the MID and 50% successful rate in inhibition trials in the SST). As a passive viewing task, there is no performance measure for the EFT.

Acquisition

Imaging type(s)	BOLD functional signal
Field strength	3 Tesla
Sequence & imaging parameters	Structural and functional MRI data were acquired at eight IMAGEN assessment sites with 3T MRI scanners of different manufacturers (Siemens, Philips, General Electric, Bruker). The scanning variables were specifically chosen to be compatible with all scanners. The same scanning protocol was used in all sites. In brief, high-resolution T1-weighted 3D

structural images were acquired for anatomical localization and co-registration with the functional time-series. Blood-oxygen-level-dependent (BOLD) functional images were acquired with gradient-echo, echo-planar imaging (EPI) sequence. For all fMRI tasks, 300 volumes were acquired for each participant, and each volume consisted of 40 slices aligned to the anterior commission/posterior commission line (2.4 mm slice thickness, 1 mm gap). The echo-time (TE) was optimized (TE=30 ms, repetition time (TR)=2,200 ms) to provide reliable imaging of subcortical areas.

Area of acquisition: Whole brain scan

Diffusion MRI: ☐ Used ☒ Not used

Preprocessing

Preprocessing software: Functional MRI data were analysed with SPM8 (Statistical Parametric Mapping, http://www.fil.ion.ucl.ac.uk/spm). Spatial preprocessing included: slice time correction to adjust for time differences due to multi-slice imaging acquisition, realignment to the first volume in line, non-linearly warping to the MNI space (based on a custom EPI template (53x63x46 voxels) created out of an average of the mean images of 400 adolescents), resampling at a resolution of 3x3x3 mm3 and smoothing with an isotropic Gaussian kernel of 5 mm full-width at half-maximum.

Normalization: see above

Normalization template: see above

Noise and artifact removal: At the first level of analysis, changes in the BOLD response for each subject were assessed by linear combinations at the individual subject level, for each experimental condition (e.g. reward anticipation high gain of Monetary Incentive Delay (MID) task), each trial was convolved with the hemodynamic response function to form regressors that account for potential noise variance, e.g. head movement, associated with the processing of reward anticipation. Estimated movement parameters were added to the design matrix in the form of 18 additional columns (three translations, three rotations, three quadratic and three cubic translations, and every three translations with a shift of ±1 TR).

Volume censoring: N/A

Statistical modeling & inference

Model type and settings: At the first level of analysis, we estimated the condition-specific functional connectivity with weighted GLM (wGLM) method. We get these condition-specific functional connectivity matrices, including reward positive feedback, reward negative feedback and reward anticipation of monetary incentive delay (MID) task; stop failure, stop success and go wrong of stop-signal task (SST); angry and neutral of emotional face task (EFT). At the second level of analysis, we used the Pearson correlation test to estimate the association between task-based functional connectivity and behavioural measurements.

Effect(s) tested: The T-Test was used to measure the strength of functional connectivity.

Specify type of analysis: ☐ Whole brain ☐ ROI-based ☒ Both

Anatomical location(s): A 268-node functional brain atlas was used (doi: 10.1016/j.neuroimage.2013.05.081.)

Statistic type for inference (See Eklund et al. 2016): The connectome-based predictive model was used to estimate the associations of whole-brain functional connectivity with externalising and internalising symptoms.

Correction: Either permutation, false discover rate correction and Bonferroni correction was applied wherever applicable.

Models & analysis

n/a	Involved in the study
☐	☒ Functional and/or effective connectivity
☒	☐ Graph analysis
☐	☒ Multivariate modeling or predictive analysis

Functional and/or effective connectivity: Pearson correlation

Multivariate modeling and predictive analysis: First, functional connectivity (FC) was calculated for each task condition per individual using the CONN toolbox. Second, at each task condition, the FC was used to predict different psychiatric disorder scores (i.e. the sum of corresponding symptoms) with the connectome-based predictive model. Finally, the brain signatures (i.e. the contributing FC) of each externalising and internalising disorder were identified and utilized to construct the general neuropsychopathological factor (i.e. the NP factor)
